# An Emerging Bacterial Leaf Disease in Rice Caused by *Pantoea ananatis* and *Pantoea eucalypti* in Northeast China

**DOI:** 10.3390/microorganisms13061376

**Published:** 2025-06-13

**Authors:** Guohua Duan, Xin Liu, Shaoqi Zhang, Mengzhu Chai, Zhao Peng, Zihan Lin, Dayong Li, Wenxian Sun

**Affiliations:** 1 College of Plant Protection, Jilin Provincial Key Laboratory of Green Management of Crop Pests and Diseases, Jilin Agricultural University, Changchun 130118, China; ghduan1990@163.com (G.D.); 15543685979@163.com (X.L.); z18863653127@163.com (S.Z.); mengzhuchai@jlau.edu.cn (M.C.); zpeng21@jlau.edu.cn (Z.P.); linzihan040510@163.com (Z.L.); 2 Department of Plant Pathology, The Ministry of Agriculture Key Laboratory of Pest Monitoring and Green Management, China Agricultural University, Beijing 100193, China

**Keywords:** rice, *Pantoea*, pathogen identification, genome

## Abstract

Rice production faces new challenges from emerging diseases due to intensive cultivation practices and climate warming in China. A new rice leaf bacterial disease has recently occurred in Northeast China. The symptoms of the disease are similar to those of bacterial leaf blight. Disease lesions spread along leaf edges and are later dried up due to water loss. In this study, 17 bacterial isolates were identified as the causal agents of the new disease following Koch’s postulates. These strains are categorized into two groups based on colony morphology and molecular characterization. Phylogenetic analysis using the five housekeeping genes *leuS*, *gyrB*, *fusA*, *pyrG*, and *rplB* reveals that the two groups of the isolates belong to *Pantoea ananatis* and *P. eucalypti*, respectively. The new rice disease is caused by *P. ananatis*, *P. eucalypti*, or a combination of both bacterial species. A complete genome map has also been assembled for *P. eucalypti*. Meanwhile, some important virulence factors have been predicted based on gene annotation and determination of extracellular enzymes. Collectively, this study represents the first report of a new rice leaf disease caused by *P. eucalypti* and the first high-quality genome assembly of *P. eucalypti* that infects rice leaves.

## 1. Introduction

Rice (*Oryza sativa*), one of the major cereal crops, feeds more than half of the world’s population. However, rice production is threatened by a variety of diseases caused by fungi, bacteria, viruses, and nematodes [1,2,3]. In recent years, a newly emerging disease has been identified in multiple countries worldwide, causing leaf necrosis and drying, grain discoloration, and plant wilting [4,5]. This disease has been successively reported in various provinces in China, including Sichuan, Zhejiang, Guangdong, and Anhui. Notably, this disease is characterized by rapid spread and significant yield losses in the epidemic regions, with symptoms similar to those of bacterial leaf blight. It has been reported that the bacterial disease is caused by *Pantoea ananatis*, *P. agglomerans*, and other species in the genus *Pantoea*, rather than *Xanthomonas oryzae* [6,7,8]. Notably, the disease is also caused by a combination of *P. ananatis* and *Enterobacter asburiae* [9,10,11]. Therefore, the causal agents of this new bacterial disease are complex and might exhibit regional variations, thus increasing difficulty and challenges in the prevention and control of the disease.

Some *P. ananatis* strains are capable of infecting various plant species, including LMG20103 isolated from *Eucalyptus* [12], LCFJ-001 from mulberry roots [13], and OC5a from onion [14], whereas other *P. ananatis* strains serve as biocontrol agents, endophytic bacteria, and opportunistic pathogens. For instance, the *P. ananatis* strain R100 isolated from rice seeds exhibits strong antagonism against rice pathogens, such as *Acidovorax avenae* subsp. *avenae* [15]. The reported endophytic strains include *P. ananatis* B1-9, an endophytic rhizobacterium capable of promoting plant growth and enhancing crop yield [16], and the *Lstr* strain identified from rice planthopper *Laodelphax striatellus* [17]. Interestingly, the LMG 5342 strain isolated from a human wound is an opportunistic pathogen [18]. These characteristics of *Pantoea* species make them an ideal model to study the evolution of endophytic bacteria, opportunistic pathogens, and plant pathogens.

The genomes of an increasing number of *Pantoea* strains from diverse ecological niches have been sequenced. The genomes of *Pantoea* strains from diverse hosts facilitate studying its genetic diversity. The complete genome sequence has been available for the pathogenic *P. ananatis* strain TZ39, causing a new rice bacterial blight disease in China [11], and also for three other rice pathogenic strains, ARC272, ARC310, and ARC311 [19]. Similarly, the whole genomes of *P. eucalypti* LMG 24197 isolated from diseased leaves of *Eucalyptus* and the endophytic *P. eucalypti* FBS135 isolated from *Pinus massoniana* have been sequenced and assembled [20,21]. These genomic resources collectively offer a comprehensive foundation for exploring the host adaptation of *Pantoea*.

Comparative genomics uncovers genomic plasticity that drives *P. ananatis* pathogenicity in diverse hosts and its adaptability to various niches [22,23]. The pathogenic mechanisms of *Pantoea* species are diverse, enabling this genus of bacteria to adapt to and interact with a wide range of hosts. The Type II and III secretion systems (T2SS and T3SS) are often tightly associated with the virulence of phytopathogenic bacteria [24,25]. Interestingly, recent studies have identified *P. ananatis* as an unusual type of Gram-negative bacteria that generally lacks T2SS and T3SS [22,26]. Instead, *P. ananatis* employs the Type VI secretion system (T6SS) for pathogenicity. Besides secretion systems, *P. ananatis* pathogenicity is also significantly influenced by virulence-associated gene clusters. The “*HiVir*” (High Virulence) gene cluster has been identified to be critical for onion pathogenicity through comparative genomics and mutational analyses [27,28]. Another key virulence gene cluster “*alt*” (Allicin tolerance), a plasmid-borne cluster, confers tolerance to thiosulfinates in *P. ananatis*, enabling its colonization of the thiosulfinate-rich environment in necrotic onion bulbs [26,29,30]. Pan-genome-wide association studies of *P. ananatis* have further revealed 28 novel genes and 1182 horizontal gene transfer events, which are linked to the acquisition of pathogenicity and virulence [31]. In addition, motility-related genes contribute to *P. ananatis* pathogenicity. Specifically, flagellum-associated proteins FlgK and MotA facilitate localization and attachment to onion surfaces, while the type IV pilus proteins PilA and PilT promote surface spreading, as demonstrated in *P. ananatis* LMG 20103 mutants [23].

In this study, we isolated the causal agents responsible for a rice leaf disease that has newly emerged in Heilongjiang and Jilin Provinces following Koch’s postulates. Phylogenetic analysis using a concatenated alignment of five housekeeping genes identified them as *P. ananatis* and *P. eucalypti* isolates and revealed their evolution relationships among *Pantoea* species. In addition, the high-quality genome of *P. eucalypti* was assembled using Nanopore long-read sequencing technology, and pathogenicity-related genes and secretion systems were further annotated. The complete genome is of great significance for the elucidation of molecular mechanisms underlying *P. eucalypti* pathogenicity.

## 2. Materials and Methods

### 2.1. Pathogen Isolation and Purification

The diseased leaves were collected from the rice variety Muyudao 78 in Jiamusi and Mudanjiang, Heilongjiang Province, China in 2019, and from the rice varieties Lijiangxintuanheigu and Jijing 88 in Meihekou City, Jilin Province in 2020. The bacterial isolates JMS78-1 and GY78-10 were isolated from diseased lesions of Muyudao 78 in Jiamusi and Mudanjiang, respectively.

The pathogens were isolated through a conventional tissue separation approach [32]. Diseased rice leaves were collected in a paddy field and were then rinsed with sterile water. Leaf slices were clipped from diseased lesions and were sterilized using 75% ethanol for 2 min. After being rinsed with sterile water three times, the slices were immersed in 1 mL of sterile water for 10 min. The leachate was diluted 100 times with sterile water and spread on LA medium (5 g yeast powder, 10 g peptone, 10 g sodium chloride, and 15 g agar per liter) plates. The plates were incubated at 28 °C for 2 d and the colonies were individually streaked onto new LA medium plates. The pure cultures from individual colonies were then stored for further studies.

### 2.2. Pathogen Identification by Koch’s Postulates

The one-month-old seedlings of the rice variety Muyudao78 were inoculated by the clipping method [33]. Briefly, the leaf tips were cut using a pair of sterilized scissors after the scissors were dipped into bacterial cultures (OD_600_ = 0.8). At least 15 leaves were inoculated for each isolate. The inoculation assays were independently repeated three times. LB medium was inoculated as a mock control. The inoculated seedlings were kept in an environmentally controllable growth room at 28 °C ± 2 °C and 90% ± 10% relative humidity for 2 d and were then moved into the greenhouse. The disease lesions and symptoms were observed and photographed at 2 weeks post inoculation. The pathogens were then isolated and identified from the disease lesions on inoculated leaves following Koch’s postulates [34].

### 2.3. Pathogen Identification by Multilocus Sequence Analysis (MLSA)

The bacterial isolates were grown in LB medium to logarithmic growth stage (OD_600_ = 0.8) and were then collected for DNA isolation. Genomic DNA was extracted using a BioFlux bacterial genomic DNA extraction kit (TransGen Biotechnology Co., Ltd., Beijing, China). Five housekeeping genes (*leuS*, *gyrB*, *fusA*, *pyrG*, and *rplB*) were amplified using the designed primers (Table 1) [35,36,37]. PCR products were separated by 1% agarose gel electrophoresis and were then purified using a DNA extraction kit (Conway Century Biotechnology Co., Ltd., Beijing, China) before sequencing [38]. The obtained sequences were used for the phylogeny and identification of *Pantoea* species.

### 2.4. Phylogenetic Tree Construction

The sequences of five housekeeping genes from different subspecies of *Pantoea* were used to construct the phylogenetic trees using Bayesian inference (BI) and maximum likelihood (ML) methods with MrBayes v3.2.6 [39] and IQ-TREE v1.6.12 [40] programs, respectively. The ModelFinder program was employed to select the best-fit model of nucleotide substitutions for each gene before tree construction. To obtain phylogenetic relationships, aligned sequences were run for 2,000,000 generations until the standard deviation of split frequencies was below 0.01 through MrBayes. Concurrently, ML analyses were conducted for 1000 bootstrap replicates using IQ-TREE. *Tatumella punctata*, *T. citrea*, and *T. terrea* were used as outgroups for the phylogenetic analysis [40].

### 2.5. Detection of Extracellular Enzymes

The activities of extracellular enzymes were determined as described previously [9]. Briefly, the activities of extracellular proteases, pectate lyases, and celluloses were determined on protease assay plates (the 1:1 mixture of LB medium and 40% skim milk, 10 g agar), pectate lyase assay plates (100 mM Tris-Cl, pH 8.5, 0.38 μM CaCl_2_, 10 g yeast powder, 10 g polygalacturonic acid, 8 g agar), and cellulose assay plates (25 mM Na_3_PO_4_, pH 7.0, 1 g sodium carboxymethyl cellulose, 8 g agar), respectively. The wells (5 mm diameter) were punched on the medium plates, and 20 μL of bacterial cells (OD_600_ = 1.0) were added into the wells. The protease assay plates were directly observed after incubation for 24 h at 28 °C. The cellulose assay plates were stained with 0.1% Congo red for 10–15 min after 14 h incubation, followed by decolorization with 1 M NaCl solution 2–3 times for 10–15 min. After 14 h incubation at 28 °C, the pectate lyase assay plates were observed after staining with 1 M HCl for color development [9].

### 2.6. Whole Genome Sequencing and Gene Annotation

The genome of GY78-10 was sequenced through the Nanopore technology using a MinION sequencer (Biomarker Biotechnology Co. Ltd., Beijing, China). Raw sequencing data were subject to quality control, and the adapters, short (<2000 bp) and low-quality (Q value ≥ 20) fragments were removed, resulting in a set of high-quality clean sequences. The clean reads were assembled using Canu v1.5 [41], and the assembly was corrected using Racon v3.4.3 [42]. Chromosome circularization and correction of the genome-starting position were performed using Circlator v1.5.5 to obtain a more accurate genome [43]. The predicted genes were functionally annotated through searching against multiple databases, including NCBI (The National Center for Biotechnology Information) non-redundant (Nr) protein, evolutionary genealogy of genes: Non-supervised Orthologous Groups (eggNOG), Kyoto Encyclopedia of Genes and Genomes (KEGG), *Swiss*-*Prot*protein database (Swiss-Prot), Gene Ontology (GO), Protein Family (Pfam), Pathogen-Host Interactions (PHI), Carbohydrate Active Enzymes (CAZy), Transporter Classification Database (TCDB), Comprehensive Antibiotic Resistance Database (CARD), Virulence Factor Database (VFDB). The GenoVi v0.2.1 program was employed to visualize predicted genomic features such as tRNA, rRNA, repetitive sequences, GC content, and gene functional information on the genome [44]. The genome-wide average nucleotide identity was calculated using FastANI v1.33 [45]. A phylogenetic tree was constructed using OrthoFinder v2.5.5 software based on single-copy orthologs [46].

### 2.7. Pathogenicity-Associated Component Analysis

Bacterial secretion systems (types I–VI) were identified using MacSyFinder v2 [47]. The genomic islands were predicted using the IslandPath-DIMOB v1.0.0 [48], and pathogenicity-related genes were identified by searching against the PHI database [49]. The secondary metabolite biosynthesis gene clusters were screened using the bacterial version of antiSMASH 6.0 [50].

## 3. Results

### 3.1. Incidence and Symptoms of a New Rice Bacterial Disease

**Figure 1:**
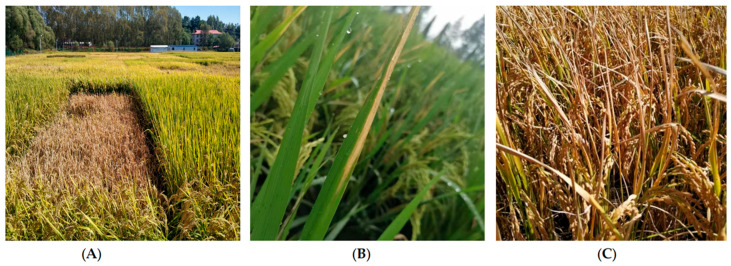
Symptoms of a new rice disease occurred in paddy fields in Northeast China. (**A**) Plant wilting caused by a new disease in paddy fields. (**B**) Disease symptoms on rice leaves at the early infection stage. (**C**) Disease symptoms on rice leaves at the late infection stage.

A new rice disease occurred seriously in paddy fields in Jiamusi and Mudanjiang cities, Heilongjiang Province in 2019, causing plant wilting in large areas (Figure 1A). This disease was found in Meihekou and Da’an City, Jilin Province in 2022 and 2024, respectively. At the early stages of the disease, the leaf tip starts to show water-soaked lesions. The lesions gradually expand downwards along the edge of the leaf. Later, the leaf edges become dry and light gray (Figure 1B). As the disease progresses to later stages, the entire leaf curls up and dries out. Certain stems may also rot. Sometimes, pale yellow and tasteless bacterial ooze may be visible at the stem base. However, no severe discoloration of the seeds has been observed yet (Figure 1C). The disease symptoms suggest that this disease is likely caused by certain type(s) of bacteria.

### 3.2. Identification of the Causal Agent Based on Koch’s Postulates

We identified the causal agent(s) from diseased leaves using the conventional tissue separation method. A total of 37 individual and representative colonies were selected when the isolates were cultured on the plates after serial dilutions. For the pathogenicity test, 37 bacterial isolates were individually inoculated into the leaves of the rice cultivar Muyudao78. Among these, 17 isolates were capable of causing visible disease lesions at 7 days post inoculation (dpi). The disease symptoms were then gradually expanded and became similar to those we observed in the field at 14 dpi. The pathogenic bacteria were isolated from the inoculated diseased leaves. The colonies of isolated bacteria were identical to those of the original pathogenic bacteria. According to Koch’s postulates, the 17 isolates were identified as pathogenic bacteria of the new rice disease and were named as follows: JMS78-1 to JMS78-4 from Jiamusi; GY78-1, GY78-9, and GY78-10 from Mudanjiang; JJ88-1 to JJ88-3 and MHC-1 to MHC-2 from Meihekou; and DA-2-2 to DA-2-4, DA-3-1, and DA-3-2 from Da’an. These bacteria exhibited yellow or light-white circular colonies with smooth surfaces and neat edges (Supplementary Figure S1).

### 3.3. Identification of P. ananatis and P. eucalypti

The isolates identified by Koch’s postulates were further characterized at the molecular level. Genomic DNA was extracted from these isolates and was then used as the template for PCR amplification of 16S rDNA. The resultant products were separated by agarose gel electrophoresis and were then purified for sequencing. The obtained sequences were subject to alignment and BLAST v.2.12.0 searches against the NCBI database. The results showed that 13 out of 17 isolates had identical 16S rDNA sequences (represented by JMS78-1), which share the highest identity with those of *P. ananatis* (≥99%). The other four isolates (represented by GY78-10) had identical 16S rDNA sequences, which share the highest identity (100%) with those of *P. eucalypti* (Supplementary Table S1).

**Figure 2:**
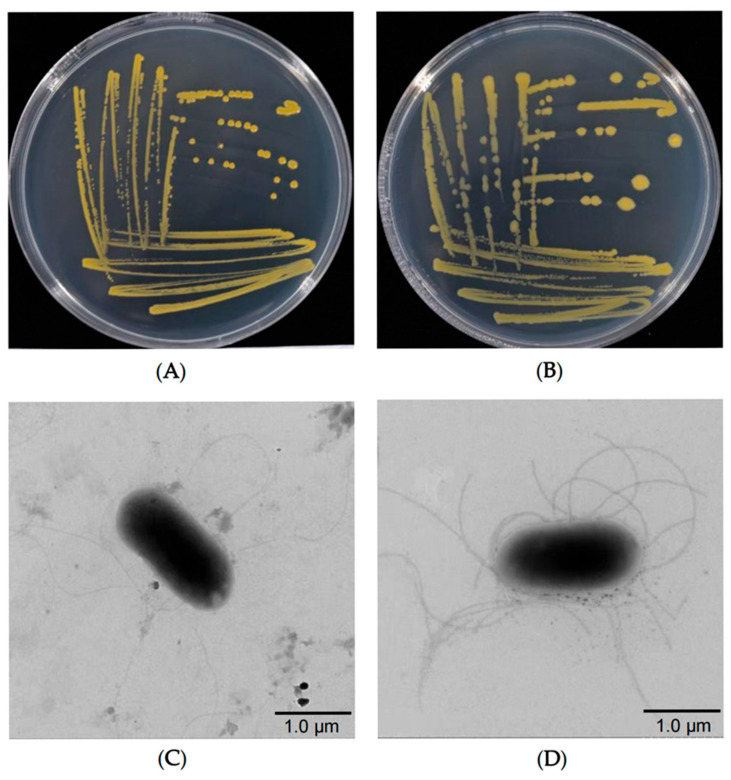
Colony and cell morphologies of the JMS78-1 and GY78-10 bacterial isolates. (**A**,**B**) Colony morphology of JMS78-1 (**A**) and GY78-10 on LA medium plates after culturing at 28 °C for 2 days (**B**). (**C**,**D**) Representative transmission electron microscope (TEM) images showing the morphology of JMS78-1 (**C**) and GY78-10 (**D**) cells (Scale bar: 1.0 μm).

**Figure 3:**
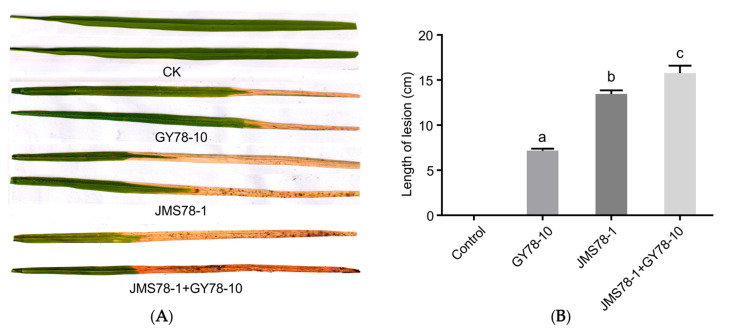
Pathogenicity assays on rice leaves for the bacterial isolates JMS78-1 and GY78-10. (**A**) Disease symptoms on the inoculated leaves by the indicated bacterial isolates. The leaves were inoculated with LB medium as a negative control. (**B**) The average length of disease lesions on the inoculated leaves caused by different bacterial isolates. Disease lesion lengths were measured at 14 dpi. The representative data from three independent experiments are shown as mean ± standard deviation (SD, *n* =  10). Lowercase letters (a–c) represent significant differences in disease lesion length caused by different bacterial isolates (*p* < 0.05).

The representative isolates JMS78-1 and GY78-10 exhibited strong pathogenicity and typical disease symptoms on rice leaves. Therefore, the two isolates were chosen for subsequent studies (Figure 2A,B). Cell morphology of JMS78-1 and GY78-10 was observed under a transmission electron microscope with a magnification of 4000×. It was found that both types of bacterial cells are rod-shaped with sizes of 1.5–2.5 μm × 0.5–1 μm and carry peritrichous flagella with about 2–5 μm in length (Figure 2C,D). Interestingly, disease lesions on the inoculated leaves caused by the mixture of JMS78-1 and GY78-10 were significantly longer than those caused by a single isolate, indicating that the two bacterial isolates have a synergistic pathogenic effect (Figure 3A,B).

**Figure 4:**
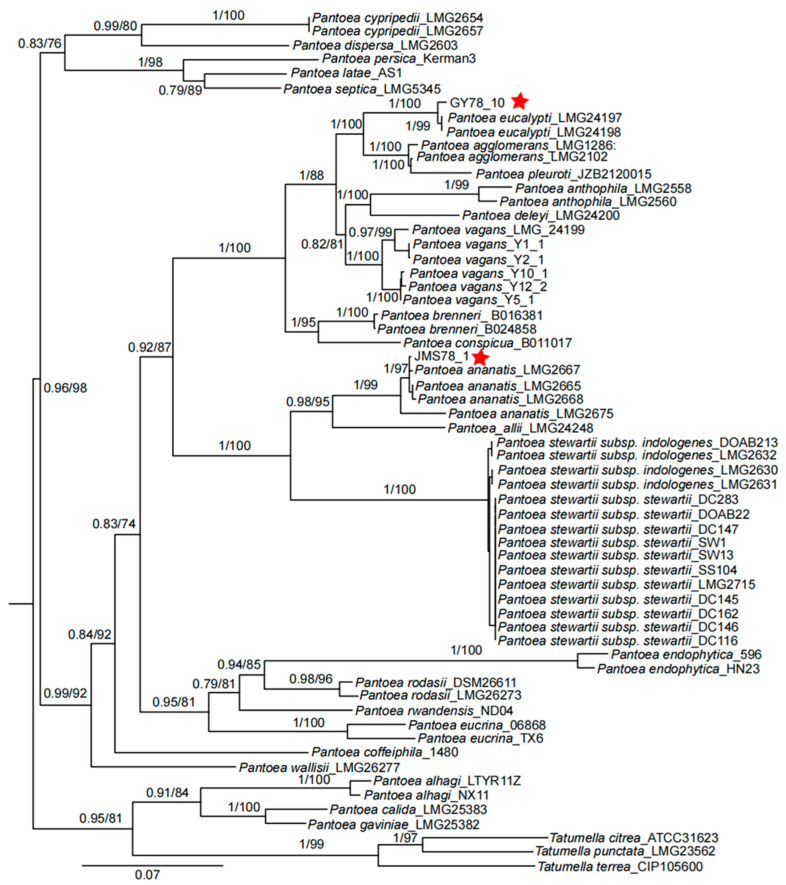
A phylogenetic tree was constructed based on the concatenated sequences of *leuS*, *gyrB*, *fusA*, *pyrG*, and *rplB* genes. The phylogenetic trees constructed through BI and ML methods were consistent. The numbers displayed at the nodes represent the posterior probabilities from the Bayesian analysis (MrBayes) and the bootstrap values based on the 1000 replicates of the ML analysis (IQ-TREE), respectively. Red stars indicate the bacterial isolate identified in this study. The scale bar represents units of substitutions per site.

To determine the evolutionary relationships of JMS78-1 and GY78-10 with other *Pantoea* species, the *leuS*, *gyrB*, *fusA*, *pyrG*, and *rplB* genes were amplified and were then sequenced. Based on these gene sequences of different *Pantoea* species, a phylogenetic tree was constructed using the concatenation method. The phylogenetic tree showed that JMS78-1 belongs to the same clade as *P. ananatis,* suggesting that it is genetically closest to *P. ananatis*. By contrast, the isolate GY78-10 is phylogenetically closest to *P. eucalypti* (Figure 4).

### 3.4. Genome Sequencing, Assembly, and Functional Annotation

**Figure 5:**
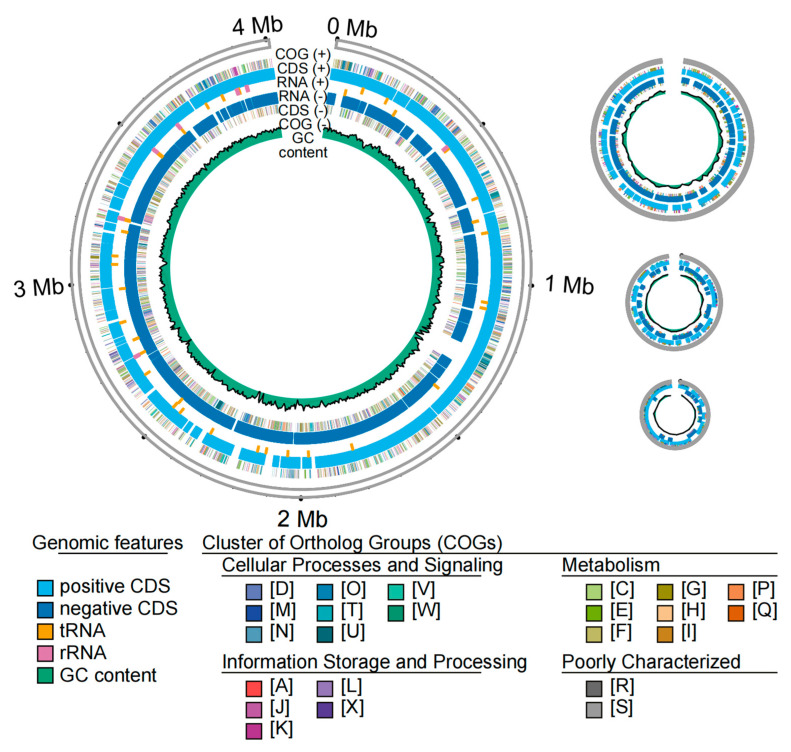
The genome and plasmid maps and functional annotation of *P. eucalypti* GY78-10. The circles from outside to center: Genomic size representation; COG (+), COG functional categories of CDSs on the positive strand; rRNA, tRNA on the positive strand; CDSs, rRNA, tRNA on the negative strand; COG (−), COG functional categories of CDSs on the negative strand; GC content. Detailed information and corresponding gene numbers in distinct functional categories in COGs indicated by different uppercase letters are provided in Supplementary Table S4. rRNA: ribosomal RNA; tRNA: transfer RNA.

The genome of the GY78-10 isolate was sequenced, generating 903,572,889 bp of clean and high-quality reads after removing the adapters, low-quality and short fragments (<2000 bp). The clean reads had an N50 length of 19,192 bp. One complete chromosomal genome with no gap, along with three plasmids, was assembled with a GC content of 54.35%. The genomic size, gene number, and GC content of three plasmids are summarized in Supplementary Table S2. The whole genome contains 4,757,181 bp, including a chromosome length of 4,008,345 bp. According to the genome assembly, 4587 genes were predicted, including 4378 protein-coding genes, 2 pseudogenes, 22 ribosomal RNA, 78 transfer RNA, and 107 other non-coding RNAs (Table 2; Figure 5). Comparative genomics revealed genomic characteristics of *P. ananatis*, *P. eucalypti*, and *Xoo* (Supplementary Table S3). In addition, the *P. eucalypti* isolate GY78-10 shares a whole-genome average nucleotide identity (ANI) of 99.1683% with the reference *P. eucalypti* strain LMG24197. Consistently, a phylogenetic tree constructed using single-copy orthologs from the genomes of 14 *Pantoea* species and the isolate GY78-10 revealed the closest phylogenetic relationship between GY78-10 and *P. eucalypti* strain LMG24197 (Supplementary Figure S2).

The genome of *P. eucalypti* encodes diverse secretion systems, including a complete Type I secretion system composed of 3 proteins, an incomplete flagellum-associated Type III secretion system (T3SS) comprising 11 proteins, one defective Type IV system with 9 proteins, one defective Type V system with 1 protein, and two complete duplicate Type VI systems each containing 14 proteins across two loci (Table 3 and Supplementary Table S5). Interestingly, no protein was predicted for the Type II secretion system or Hrp-associated T3SS. In addition, 11 genomic islands were predicted to contain 7 to 44 genes. Through searching against the PHI database, 592 potential pathogenicity-related genes with an identity of ≥50% and a coverage of ≥30% were identified (Table 3). Comparative analyses were performed to compare different types of secretion systems and pathogenicity-related genes among *P. ananatis*, *P. eucalypti*, and *Xoo* (Supplementary Tables S6 and S7). Through genomic analysis and searching against the antiSMASH 6.0 database, 3 secondary metabolite gene clusters were identified to be related to the biosynthesis of arylpolyene, terpene, and NI-siderophore.

### 3.5. Comparative Analysis of Virulence Factors

**Figure 6:**
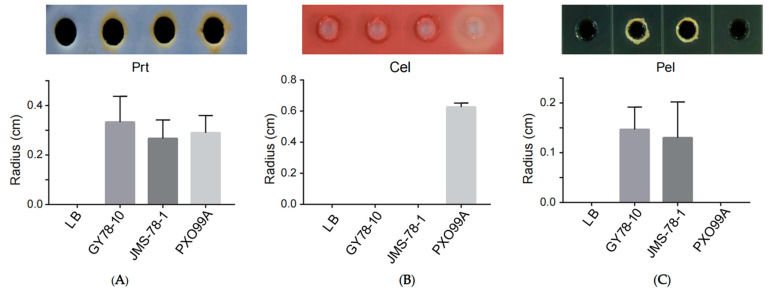
The ability to secrete different extracellular enzymes in *P. eucalypti* GY78-10, *P. ananatis* JMS-78-1, and *X. oryzae* pv. *oryzae* PXO99A. (**A**–**C**) Detection of extracellular proteases (Prt), cellulases (Cel), and pectate lyases (Pel) in GY78-10, JMS-78-1, and PXO99A strains on culturing plates. Upper panels, Prt images were captured at 24 h after culturing, while Pel and Cel images were captured at 14 h after culturing. Lower panels, the radius of the clear area caused by extracellular enzymes. Data are shown as mean  ± SD (*n* = 3). Bacterial cultures (20 μL, OD_600_ = 1.0) were loaded onto assay plates. All the plates were incubated at 28 °C and stained for visibility. Radius of the inhibition zone was measured.

To investigate the pathogenicity mechanism of the isolated pathogens, the secretion of extracellular enzymes was detected in *P. eucalypti* GY78-10, *P. ananatis* JMS-78-1, and the bacterial blight pathogen *X. oryzae* pv. *oryzae* (*Xoo*) PXO99A. The results showed that both GY78-10 and JMS-78-1 produced extracellular proteases, and similarly, extracellular protease was also detectable in PXO99A [51] (Figure 6A). By contrast, *X. oryzae* pv. *oryzae* PXO99A secreted a great amount of cellulases, while the GY78-10 and JMS-78-1 isolates did not produce any cellulases (Figure 6B). In addition, both GY78-10 and JMS-78-1 produced pectate lyases under our testing conditions (Figure 6C).

## 4. Discussion

Rice commercial production is seriously threatened by several important diseases, such as fungal blasts and bacterial leaf blight. Moreover, some newly emerging rice diseases cause severe yield losses on a regional scale. The bacterial disease caused by *P. ananatis* was first reported in rice more than ten years ago and was successively found in different regions in China [9]. *E. asburiae* has also been identified as a causal agent of this leaf bacterial disease in China, with *P. ananatis* occasionally acting as a co-infection pathogen [9]. The findings indicate that the causal agents of this leaf bacterial disease are complex.

In this study, pathogenic bacteria causing an emerging rice leaf disease in Heilongjiang and Jilin Provinces have been identified as *P. ananatis* and *P. eucalypti*. First, multiple bacterial isolates were isolated from the diseased leaves and were then confirmed to be causal agents following Koch’s postulates. Furthermore, to determine their taxonomic positions, phylogenetic analyses were performed using the sequences of 16S rDNA, the concatenated sequences of five housekeeping genes (*leuS*, *gyrB*, *fusA*, *pyrG*, and *rplB*), and genome-wide single-copy orthologs. The consistent results showed that one group of isolated strains was classified as *P. ananatis*, while the other was identified as *P. eucalypti*. Knowledge is very limited about *P. eucalypti* as a pathogenic bacterium. It has been previously reported that *P. eucalypti* causes wilting and dieback symptoms on the leaves and branches of eucalyptus trees in Uganda, Uruguay, and Argentina, and brown stem rot disease in maize in South Africa [52]. To our knowledge, this study represents the first report that *P. eucalypti* causes rice leaf disease.

*P. ananatis* is a well-known plant pathogen. It was first isolated from pineapple in the Philippines [53] and was later found to infect a variety of plant species, causing pineapple fruit rot [54], onion rot [14], maize brown stalk rot [14], and Eucalyptus leaf blight [55]. *Pantoea* species are recognized as plant pathogens that cause economically significant diseases in agriculturally important crops, including maize [56], onion, rice [57], sorghum [58], and wheat [59]. *P. ananatis* was first found to cause rice disease in Australia [4]. Since then, it has been subsequently reported throughout the major rice-growing countries worldwide, such as India, South Korea [6], Russia [5], and China [11].

To provide an insight into the pathogenicity mechanisms of *P. eucalypti*, we assembled the complete genome of the rice pathogenic *P. eucalypti* isolate GY78-10. Together with other publicly available genomes of the *Pantoea* species, the *P. eucalypti* genome offers valuable genetic resources for further exploration of the pathogenicity mechanisms and evidence of host adaptation within the *Pantoea* genus in rice. T6SS plays a critical role in the pathogenicity of *P. ananatis* toward onion plants and contributes to bacterial competition [60,61]. Notably, we identified duplicate gene clusters encoding T6SS at two distinct genomic loci in the assembled *P. eucalypti* genome, further suggesting the importance of T6SS in the pathogenicity of the *P. eucalypti* isolate GY78-10. Unlike most Gram-negative plant pathogenic bacteria, *P. ananatis* does not rely on T2SS, which is important for secreting cell wall-degrading enzymes, or T3SS for delivering virulence effectors into host cells [62]. Consistently, only flagellum-related T3SS, but not Hrp-associated T3SS, is present in the *P. eucalypti* GY78-10 genome. In contrast, T3SS is essential for *P. agglomerans* pathogenicity [63]. In addition to secretion systems, pathogenicity-related gene clusters greatly contribute to *Pantoea* infection in various hosts. Through sequence similarity searches, the genome of *P. eucalypti* GY78-10 was found to lack some identified pathogenicity-related gene clusters, such as *Halophos* in *P. stewartii* [64] and *alt* and *HiVir* in *P. ananatis* [27,30,65]. These results further highlight the diversity of pathogenic mechanisms in *Pantoea* species.

Extracellular enzymes including cellulases, pectate lyases, and proteases play varying roles in the pathogenicity of plant pathogens. For instance, extracellular cellulases are essential virulence factors in pathogens such as *Clavibacter michiganensis* [66] and *Xoo* [67]. In our study, cellulases were detected in *Xoo* but not in *P. ananatis* or *P. eucalypti*, indicating that the secreted cellulases contribute to the virulence of some bacteria, but not to the virulence of other bacterial species. By contrast, the activity of pectate lyases was detected in *P. eucalypti* GY78-10 and *P. ananatis* JMS-78-1, but was not detected in *Xoo*, suggesting that pectate lyases are not universally required for virulence in the bacterial species. Extracellular proteases that break host barriers and enhance virulence [68,69] were detected in *P. eucalypti* GY78-10 and *P. ananatis* JMS-78-1. These results demonstrate the differential distribution of these enzymes among *Pantoea* species and *Xoo*, emphasizing their variable contributions to pathogenicity. This diversity in extracellular cellulases, pectate lyases, and proteases in different bacteria indicates the diverse and adaptive pathogenic strategies of plant-associated bacteria.

Although *P. eucalypti* and *P. ananatis* belong to distinct phylogenetic clades with a significant genetic divergence (Figure 4), both are capable of infecting rice leaves. Similar phenomena have been observed in other *Pantoea* species. *P. agglomerans* and *P. ananatis* can both infect maize (*Zea mays*) and onion (*Allium cepa*) despite their significant genetic divergence. Likewise, *P. stewartii* and *P. dispersa* also infect overlapping plant hosts despite their genetic distance [52]. The ability of genetically distant *Pantoea* species to infect the same hosts demonstrates the remarkable adaptability of this genus. This adaptability highlights the ecological flexibility of the genus and provides valuable insights into host-pathogen interactions and the evolutionary dynamics of *Pantoea* species.

## 5. Conclusions

The rice leaf bacterial disease caused by *P. ananatis* and *P. eucalypti* is newly emerged in Northeast China, indicating that the novel bacterial disease has spread to the cold temperate zone. Climate change, such as warmer and more humid environments in Northeast China, may facilitate the occurrence of the rice leaf bacterial disease. The infection processes and pathogenic mechanisms of *P. ananatis* and *P. eucalypti* in causing rice diseases remain largely unknown. Understanding the diversity and accurately identifying the causal agents are crucial for disease management and the development of effective control strategies. Further efforts should be made to in-depth characterize the *P. ananatis* and *P. eucalypti* strains causing this disease, including the specific virulence determinants and mechanisms that will contribute to disease resistance breeding and the molecular design of pesticides.

## Figures and Tables

**Table 1:** The primers used in this study.

Gene	Primer	Primer Sequence	Template Size (bp)	Reference
*FusA*	fusA3	5′-CATCGGTATCAGTGCKCACATCGA-3′	588	[37]
	fusA4	5′-CAGCATCGCCTGAACRCCTTTGTT-3′		
*leuS*	leuS3	5′-CAGACCGTGCTGGCCAACGARCARGT-3′	643	[36]
	leuS4	5′-CGGCGCGCCCCARTARCGCT-3′		
*pyrG*	pyrG3	5′-GGGGTCGTATCCTCTCTGGGTAAAGG-3′	306	
	pyrG4	5’GGAACGGCAGGGATTCGATATCNCCKA-3’		
*rplB*	rplB3	5’-CAGTTGAACGTCTTGAGTACGATCC-3’	333	
	rplB4	5’-CACCACCACCATGYGGGTGRTC-3’		
*gyrB*	gyrB3	5GCGTAAGCGCCCGGGTATGTA-3’	722	
	gyrB4	5’-CCGTCGACGTCCGCATCGGTCAT-3’		

**Table 2:** The features of genome assembly of *P. eucalypti* isolate GY78-10.

Genomic Features	GY78-10
Total length of clean reads (bp)	903,572,889
GC content (%)	54.35
Chromosome	1
Chromosome length (bp)	4,008,345
Plasmids	3
Total genes	4587
Total protein-coding genes	4378
Pseudogenes	2
Ribosomal RNA genes	22
Transfer RNA genes	78
Other non-coding RNA genes	107

**Table 3:** Pathogenicity-associated components in the genome.

Features	Name	Number	Component Number	Core Structure
Secretion System	T1S	1	3	complete
	T2S	0	0	lost
	T3S (Flagellar)	1	11	defective
	T4S	1	9	defective
	T5S	1	1	defective
	T6S	2	14	complete/2 Locus
Genomic island		11	27/13/18/10/9/30/14/44/7/14/27	
Pathogenicity			350	
Secondary metabolite biosynthesisgene clusters	arylpolyene	1	61	
	terpene	1	28	
	NI-siderophore	1	26	

## Data Availability

The genome sequence of *P. eucalypti* strain GY78-10 has been deposited in the NCBI GenBank under BioProject number PRJNA1262631 with accession number CP194016. The sequences of five housekeeping genes (*leuS, gyrB, fusA, pyrG,* and *rplB*) have been deposited in the NCBI GenBank under accession numbers PV738888-PV738897. Data are contained within the article or Supplementary Materials.

## Supplementary Materials

The following Supporting Information can be downloaded at: https://www.mdpi.com/article/10.3390/microorganisms13061376/s1, Figure S1: The colony morphological characteristics of 17 isolated strains; Figure S2: The whole-genome phylogenetic analysis of isolate GY78-10 and 14 other *Pantoea* species; Table S1: Bacterial isolates and tentative taxonomic status according to 16S rDNA sequences; Table S2: Genomic characteristics of three plasmids; Table S3: Comparative genomic analysis of *P. ananatis*, *P. eucalypti*, and *Xoo*; Table S4: Gene counts and functional descriptions of COG categories in the *P. eucalypti* GY78-10 genome; Table S5: Genomic identification of secretion systems, pathogenicity-related genes, genomic islands, and secondary metabolite biosynthetic gene clusters; Table S6: Comparative analysis of secretion systems across *P. ananatis*, *P. eucalypti*, and *Xoo*; Table S7: Identification of virulence-related factors in *P. ananatisv* and *Xoo* through searches against the PHI database (Pathogen-Host Interactions).

## References

[1] Li D., Li S., Wei S., Sun W. (2021). Strategies to manage rice sheath blight: Lessons from interactions between rice and *Rhizoctonia solani*. Rice.

[2] Liu Z., Zhu Y., Shi H., Qiu J., Ding X., Kou Y. (2021). Recent progress in rice broad-spectrum disease resistance. Int. J. Mol. Sci..

[3] Sun W., Fan J., Fang A., Li Y., Tariqjaveed M., Li D., Hu D., Wang W.-M. (2020). *Ustilaginoidea virens*: Insights into an emerging rice pathogen. Annu. Rev. Phytopathol..

[4] Cother E.J., Reinke R., McKenzie C., Lanoiselet V., Noble D. (2004). An unusual stem necrosis of rice caused by *Pantoea ananas* and the first record of this pathogen on rice in Australia. Australas. Plant Pathol..

[5] Egorova M., Mazurin E., Ignatov A. (2015). First report of *Pantoea ananatis* causing grain discolouration and leaf blight of rice in Russia. New Dis. Rep..

[6] Choi O.-H., Kim H.-Y., Lee Y.-S., Kim J.-W., Moon J.-S., Hwang I.-G. (2012). First report of sheath rot of rice caused by *Pantoea ananatis* in Korea. Plant Pathol. J..

[7] Lee H., Hong J., Kim S. (2010). First report of leaf blight caused by *Pantoea agglomerans* on rice in Korea. Plant Dis..

[8] Mondal K., Mani C., Singh J., Kim J.-G., Mudgett M. (2011). A new leaf blight of rice caused by *Pantoea ananatis* in India. Plant Dis..

[9] Xue Y., Hu M., Chen S., Hu A., Li S., Han H., Lu G., Zeng L., Zhou J. (2021). Enterobacter asburiae and *Pantoea ananatis* causing rice bacterial blight in China. Plant Dis..

[10] Yan H., Yu S., Xie G., Fang W., Su T., Li B. (2010). Grain discoloration of rice caused by *Pantoea ananatis* (synonym *Erwinia uredovora*) in China. Plant Dis..

[11] Yu L., Yang C., Ji Z., Zeng Y., Liang Y., Hou Y. (2022). First report of new bacterial leaf blight of rice caused by *Pantoea ananatis* in Southeast China. Plant Dis..

[12] De Maayer P., Chan W.Y., Venter S.N., Toth I.K., Birch P.R., Joubert F., Coutinho T.A. (2010). Genome sequence of *Pantoea ananatis* LMG20103, the causative agent of *Eucalyptus* blight and dieback. J. Bacteriol..

[13] Yuan T., Huang Y., Luo L., Wang J., Li J., Chen J., Qin Y., Liu J. (2023). Complete Genome sequence of *Pantoea ananatis* strain LCFJ-001 isolated from bacterial wilt mulberry. Plant Dis..

[14] Asselin J.A.E., Bonasera J.M., Helmann T.C., Beer S.V., Stodghill P.V. (2021). Complete genome sequence resources for the onion pathogen, *Pantoea ananatis* OC5a. Phytopathology.

[15] Wu L., Liu R., Niu Y., Lin H., Ye W., Guo L., Hu X. (2016). Whole genome sequence of *Pantoea ananatis* R100, an antagonistic bacterium isolated from rice seed. J. Biotechnol..

[16] Kim H.J., Lee J.H., Kang B.R., Rong X., McSpadden Gardener B.B., Ji H.J., Park C.-S., Kim Y.C. (2012). Draft genome sequence of *Pantoea ananatis* B1-9, a nonpathogenic plant growth-promoting bacterium. J. Bacteriol..

[17] Bing X.-L., Wan Y.-Y., Liu H.-H., Ji R., Zhao D.-S., Niu Y.-D., Li T.-P., Hong X.-Y. (2022). Characterization of *Pantoea ananatis* from rice planthoppers reveals a clade of rice-associated *P. ananatis* undergoing genome reduction. Microb. Genom..

[18] De Maayer P., Chan W.Y., Rezzonico F., Bühlmann A., Venter S.N., Blom J., Goesmann A., Frey J.E., Smits T.H., Duffy B. (2012). Complete genome sequence of clinical isolate *Pantoea ananatis* LMG 5342. J. Bacteriol..

[19] Kini K., Lefeuvre P., Poulin L., Silué D., Koebnik R. (2020). Genome resources of three West African strains of *Pantoea ananatis* causing bacterial blight and grain discoloration of rice. Phytopathology.

[20] Song Z., Lu Y., Liu X., Wei C., Oladipo A., Fan B. (2020). Evaluation of *Pantoea eucalypti FBS135* for pine (*Pinus massoniana*) growth promotion and its genome analysis. J. Appl. Microbiol..

[21] Wei C., Song Z., Lu Y., Zhao Y., Fan B. (2021). Relationship of the Pine Growth Promoting *Pantoea eucalypti FBS135* with Type Strains *P. eucalypti* LMG 24197T and *P. vagans* 24199T. Life.

[22] De Maayer P., Chan W.Y., Rubagotti E., Venter S.N., Toth I.K., Birch P.R., Coutinho T.A. (2014). Analysis of the *Pantoea ananatis* pan-genome reveals factors underlying its ability to colonize and interact with plant, insect and vertebrate hosts. BMC Genom..

[23] Weller-Stuart T., De Maayer P., Coutinho T. (2017). *Pantoea ananatis*: Genomic insights into a versatile pathogen. Mol. Plant Pathol..

[24] Deng W., Marshall N.C., Rowland J.L., McCoy J.M., Worrall L.J., Santos A.S., Strynadka N.C., Finlay B.B. (2017). Assembly, structure, function and regulation of type III secretion systems. Nat. Rev. Microbiol..

[25] Naskar S., Hohl M., Tassinari M., Low H.H. (2021). The structure and mechanism of the bacterial type II secretion system. Mol. Microbiol..

[26] Stice S.P., Stumpf S.D., Gitaitis R.D., Kvitko B.H., Dutta B. (2018). *Pantoea ananatis* genetic diversity analysis reveals limited genomic diversity as well as accessory genes correlated with onion pathogenicity. Front. Microbiol..

[27] Asselin J.A.E., Bonasera J.M., Beer S.V. (2018). Center rot of onion (*Allium cepa*) caused by *Pantoea ananatis* requires pepM, a predicted phosphonate-related gene. Mol. Plant-Microbe Interact..

[28] Myers B.K., Shin G.Y., Agarwal G., Stice S.P., Gitaitis R.D., Kvitko B.H., Dutta B. (2023). Genome-wide association and dissociation studies in Pantoea ananatis reveal potential virulence factors affecting Allium porrum and Allium fistulosum× Allium cepa hybrid. Front. Microbiol..

[29] Stice S.P., Shin G.Y., De Armas S., Koirala S., Galván G.A., Siri M.I., Severns P.M., Coutinho T., Dutta B., Kvitko B.H. (2021). The distribution of onion virulence gene clusters among *Pantoea* spp.. Front. Plant Sci..

[30] Stice S.P., Thao K.K., Khang C.H., Baltrus D.A., Dutta B., Kvitko B.H. (2020). Thiosulfinate tolerance is a virulence strategy of an atypical bacterial pathogen of onion. Curr. Biol..

[31] Agarwal G., Choudhary D., Stice S.P., Myers B.K., Gitaitis R.D., Venter S.N., Kvitko B.H., Dutta B. (2021). Pan-genome-wide analysis of *Pantoea ananatis* identified genes linked to pathogenicity in onion. Front. Microbiol..

[32] Fang Z.D. (1998). Research Methods on Plant Pathology.

[33] Ke Y., Hui S., Yuan M. (2017). *Xanthomonas oryzae* pv. oryzae Inoculation and Growth Rate on Rice by Leaf Clipping Method. Bio-protocol.

[34] Ross L.N., Woodward J.F. (2016). Koch’s postulates: An interventionist perspective. Stud. Hist. Philos. Biol. Biomed. Sci..

[35] De Armas S., Galvan G.A., Lapaz M.I., Gonzalez-Barrios P., Vicente E., Pianzzola M.J., Siri M.I. (2022). Phylogeny and Identification of *Pantoea* Species Associated with Bulb Rot and Bacterial Leaf Blight of Onion Crops in Uruguay. Plant Dis..

[36] Delétoile A., Decré D., Courant S., Passet V., Audo J., Grimont P., Arlet G., Brisse S. (2009). Phylogeny and identification of *Pantoea* species and typing of *Pantoea agglomerans* strains by multilocus gene sequencing. J. Clin. Microbiol..

[37] Salerno A., Delétoile A., Lefevre M., Ciznar I., Krovacek K., Grimont P., Brisse S. (2007). Recombining population structure of *Plesiomonas shigelloides* (Enterobacteriaceae) revealed by multilocus sequence typing. J. Biotechnol..

[38] Wei C., Wang S., Liu P., Cheng S.-T., Qian G., Wang S., Fu Y., Qian W., Sun W. (2021). The PdeK-PdeR two-component system promotes unipolar localization of FimX and pilus extension in *Xanthomonas oryzae* pv. oryzicola. Sci. Signal..

[39] Ronquist F., Teslenko M., Van Der Mark P., Ayres D.L., Darling A., Höhna S., Larget B., Liu L., Suchard M.A., Huelsenbeck J.P. (2012). MrBayes 3.2: Efficient Bayesian phylogenetic inference and model choice across a large model space. Syst. Biol..

[40] Nguyen L.-T., Schmidt H.A., Von Haeseler A., Minh B.Q. (2015). IQ-TREE: A fast and effective stochastic algorithm for estimating maximum-likelihood phylogenies. Mol. Biol. Evol..

[41] Koren S., Walenz B.P., Berlin K., Miller J.R., Bergman N.H., Phillippy A.M. (2017). Canu: Scalable and accurate long-read assembly via adaptive k-mer weighting and repeat separation. Genome Res..

[42] Vaser R., Sović I., Nagarajan N., Šikić M. (2017). Fast and accurate de novo genome assembly from long uncorrected reads. Genome Res..

[43] Hunt M., Silva N.D., Otto T.D., Parkhill J., Keane J.A., Harris S.R. (2015). Circlator: Automated circularization of genome assemblies using long sequencing reads. Genome Biol..

[44] Cumsille A., Durán R.E., Rodríguez-Delherbe A., Saona-Urmeneta V., Cámara B., Seeger M., Araya M., Jara N., Buil-Aranda C. (2023). GenoVi, an open-source automated circular genome visualizer for bacteria and archaea. PLoS Comput. Biol..

[45] Jain C., Rodriguez-R L.M., Phillippy A.M., Konstantinidis K.T., Aluru S. (2018). High throughput ANI analysis of 90K prokaryotic genomes reveals clear species boundaries. Nat. Commun..

[46] Emms D.M., Kelly S. (2019). OrthoFinder: Phylogenetic orthology inference for comparative genomics. Genome Biol..

[47] Néron B., Denise R., Coluzzi C., Touchon M., Rocha E.P., Abby S.S. (2023). MacSyFinder v2: Improved modelling and search engine to identify molecular systems in genomes. Peer Community J..

[48] Bertelli C., Brinkman F.S. (2018). Improved genomic island predictions with IslandPath-DIMOB. Bioinformatics.

[49] Urban M., Cuzick A., Seager J., Wood V., Rutherford K., Venkatesh S.Y., Sahu J., Iyer S.V., Khamari L., De Silva N. (2022). PHI-base in 2022: A multi-species phenotype database for Pathogen-Host Interactions. Nucleic Acids Res..

[50] Blin K., Shaw S., Kloosterman A.M., Charlop-Powers Z., Van Wezel G.P., Medema M.H., Weber T. (2021). antiSMASH 6.0: Improving cluster detection and comparison capabilities. Nucleic Acids Res..

[51] Zou H.-S., Song X., Zou L.-F., Yuan L., Li Y.-R., Guo W., Che Y.-Z., Zhao W.-X., Duan Y.-P., Chen G.-Y. (2012). EcpA, an extracellular protease, is a specific virulence factor required by *Xanthomonas oryzae* pv. *oryzicola* but not by *X. oryzae* pv. *oryzae* in rice. Microbiology.

[52] Brady C.L., Venter S.N., Cleenwerck I., Engelbeen K., Vancanneyt M., Swings J., Coutinho T.A. (2009). *Pantoea vagans* sp. nov., *Pantoea eucalypti* sp. nov., *Pantoea deleyi* sp. nov. and *Pantoea anthophila* sp. nov. Int. J. Syst. Evol. Microbiol..

[53] Serrano F.B. (1928). Bacterial fruitlet brown rot of pineapples in the Philippines. Philipp. J. Sci..

[54] Adam Z., Tambong J.T., Lewis C.T., Lévesque C.A., Chen W., Bromfield E.S., Khan I.U., Xu R. (2014). Draft genome sequence of *Pantoea ananatis* strain LMG 2665T, a bacterial pathogen of pineapple fruitlets. Genome Announc..

[55] Coutinho T., Preisig O., Mergaert J., Cnockaert M., Riedel K.-H., Swings J., Wingfield M. (2002). Bacterial blight and dieback of *Eucalyptus* species, hybrids, and clones in South Africa. Plant Dis..

[56] Goszczynska T., Botha W., Venter S., Coutinho T. (2007). Isolation and identification of the causal agent of brown stalk rot, a new disease of maize in South Africa. Plant Dis..

[57] Kini K., Agnimonhan R., Afolabi O., Soglonou B., Silué D., Koebnik R. (2017). First report of a new bacterial leaf blight of rice caused by *Pantoea ananatis* and *Pantoea stewartii* in Togo. Plant Dis..

[58] Cota L., Costa R., Silva D., Parreira D., Lana U., Casela C. (2010). First report of pathogenicity of *Pantoea ananatis* in sorghum (*Sorghum bicolor*) in Brazil. Australas. Plant Pathol..

[59] Krawczyk K., Wielkopolan B., Obrępalska-Stęplowska A. (2020). *Pantoea ananatis*, a new bacterial pathogen affecting wheat plants (*Triticum* L.) in Poland. J. Pathog..

[60] Guevarra R.B., Magez S., Peeters E., Chung M.S., Kim K.H., Radwanska M. (2021). Comprehensive genomic analysis reveals virulence factors and antibiotic resistance genes in *Pantoea agglomeran*s KM1, a potential opportunistic pathogen. PLoS ONE.

[61] Shyntum D.Y., Theron J., Venter S.N., Moleleki L.N., Toth I.K., Coutinho T.A. (2015). *Pantoea ananatis* utilizes a type VI secretion system for pathogenesis and bacterial competition. Mol. Plant-Microbe Interact..

[62] Chang J.H., Desveaux D., Creason A.L. (2014). The ABCs and 123s of bacterial secretion systems in plant pathogenesis. Annu. Rev. Phytopahtol..

[63] Nissan G., Gershovits M., Morozov M., Chalupowicz L., Sessa G., Manulis-Sasson S., Barash I., Pupko T. (2018). Revealing the inventory of type III effectors in *Pantoea agglomerans* gall-forming pathovars using draft genome sequences and a machine-learning approach. Mol. Plant Pathol..

[64] Zhao M., Shin G.Y., Stice S., Bown J.L., Coutinho T., Metcalf W.W., Gitaitis R., Kvitko B., Dutta B. (2023). A novel biosynthetic gene cluster across the *Pantoea* species complex is important for pathogenicity in onion. Mol. Plant-Microbe Interact..

[65] Polidore A.L., Furiassi L., Hergenrother P.J., Metcalf W.W. (2021). A phosphonate natural product made by *Pantoea ananatis* is necessary and sufficient for the hallmark lesions of onion center rot. MBio.

[66] Hwang I.S., Oh E.-J., Lee H.B., Oh C.-S. (2019). Functional characterization of two cellulase genes in the Gram-positive pathogenic bacterium *Clavibacter michiganensis* for wilting in tomato. Mol. Plant-Microbe Interact..

[67] Tayi L., Kumar S., Nathawat R., Haque A.S., Maku R.V., Patel H.K., Sankaranarayanan R., Sonti R.V. (2018). A mutation in an exoglucanase of *Xanthomonas oryzae* pv. oryzae, which confers an endo mode of activity, affects bacterial virulence, but not the induction of immune responses, in rice. Mol. Plant Pathol..

[68] Boehm M., Hoy B., Rohde M., Tegtmeyer N., Bæk K.T., Oyarzabal O.A., Brøndsted L., Wessler S., Backert S. (2012). Rapid paracellular transmigration of *Campylobacter jejuni* across polarized epithelial cells without affecting TER: Role of proteolytic-active HtrA cleaving E-cadherin but not fibronectin. Gut Pathog..

[69] Chitlaru T., Israeli M.a., Bar-Haim E., Elia U., Rotem S., Ehrlich S., Cohen O., Shafferman A. (2016). Next-generation *Bacillus anthracis* live attenuated spore vaccine based on the htrA-(high temperature requirement A) Sterne strain. Sci. Rep..

