# Supplementary material for: An Emerging Bacterial Leaf Disease in Rice Caused by Pantoea ananatis and Pantoea eucalypti in Northeast China
Source: Microorganisms. 2025 Jun 13;13(6):1376. doi: 10.3390/microorganisms13061376 (PMC12195282; doi:10.3390/microorganisms13061376)
Supplement: Supplementary file 1 [file microorganisms-13-01376-s001.zip › Figure S2.pdf]

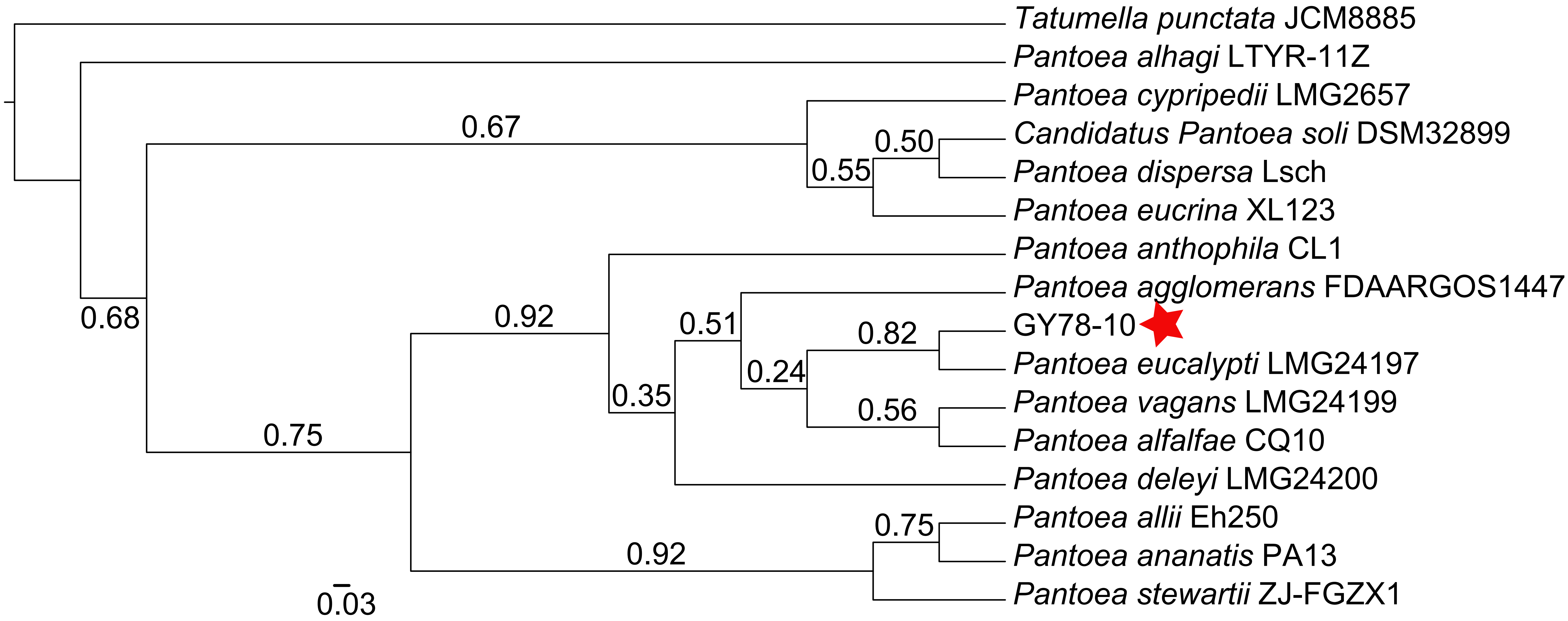


Figure S2. The whole-genome phylogenetic analysis of isolate GY78-10. The phylogenetic treewas constructed using the Orthofind program based on single copy orthologs of bacterial strains from the *Pantoea* species, with *Tatumella citrea* (Strain: DSM13699) designated as the outgroup. The red star indicates the phylogenetic position of isolate GY78-10.
