# Supplementary material for: An Emerging Bacterial Leaf Disease in Rice Caused by Pantoea ananatis and Pantoea eucalypti in Northeast China
Source: Microorganisms. 2025 Jun 13;13(6):1376. doi: 10.3390/microorganisms13061376 (PMC12195282; doi:10.3390/microorganisms13061376)
Supplement: Supplementary file 1 [file microorganisms-13-01376-s001.zip › Table S1.pdf]

Table S1: Bacterial isolates and tentative taxonomic status according to 16S rDNA sequences.

No.	Isolate	Source	Tentative taxonomic status
1	JMS78-1	Huachuan town, Jiamusi city, Heilongjiang Province	*Pantoea ananatis*
2	JMS78-2		*Pantoea ananatis*
3	JMS78-3		*Pantoea ananatis*
4	JMS78-4		*Pantoea ananatis*
5	GY78-1	Hailin town, Mudanjiang city, Heilongjiang Province	*Pantoea ananatis*
6	GY78-9		*Pantoea eucalypti*
7	GY78-10		*Pantoea eucalypti*
8	JJ88-1	Hailong Village, Meihekou city, Jilin Province	*Pantoea ananatis*
9	JJ88-2		*Pantoea ananatis*
10	JJ88-3		*Pantoea ananatis*
11	MHC-1		*Pantoea ananatis*
12	MHC-2		*Pantoea ananatis*
13	DA-2-2	Xinping'an Village, Da'an city, Jilin Province	*Pantoea ananatis*
14	DA-2-3		*Pantoea ananatis*
15	DA-2-4		*Pantoea ananatis*
16	DA-3-1		*Pantoea eucalypti*
17	DA-3-2		*Pantoea eucalypti*
