# Supplementary material for: An Emerging Bacterial Leaf Disease in Rice Caused by Pantoea ananatis and Pantoea eucalypti in Northeast China
Source: Microorganisms. 2025 Jun 13;13(6):1376. doi: 10.3390/microorganisms13061376 (PMC12195282; doi:10.3390/microorganisms13061376)
Supplement: Supplementary file 1 [file microorganisms-13-01376-s001.zip › Table S2.pdf]

Table S2 Genomic characteristics of three plasmids

	Length (bp)	Gene number	CDS number	GC%
plasmid 1	491431	482	482	52.49
plasmid 2	169775	143	143	51.1
plasmid 3	87630	97	97	50.44
