# Supplementary material for: An Emerging Bacterial Leaf Disease in Rice Caused by Pantoea ananatis and Pantoea eucalypti in Northeast China
Source: Microorganisms. 2025 Jun 13;13(6):1376. doi: 10.3390/microorganisms13061376 (PMC12195282; doi:10.3390/microorganisms13061376)
Supplement: Supplementary file 1 [file microorganisms-13-01376-s001.zip › Table S3.pdf]

Table S3 Comparative analysis of genomic features among *Pantoea ananatis*, *Pantoea eucalypti*, and *Xanthomonas oryzae* pv. *oryzae* (*Xoo*)

	P. ananatis	*Xoo*	*P. eucalypti*
Strain	PA13 (GCF_000233595.1)	PXO99A (GCF_000019585.2)	GY78-10
Chromosome length (Mbp)	4.867	5.239	4.008
Gene number	4609	4904	4587
Protein-coding genes	4428	3913	4378
Plasmid number	1	0	3
GC (%)	53.5	63.5	54.4
