# Supplementary material for: An Emerging Bacterial Leaf Disease in Rice Caused by Pantoea ananatis and Pantoea eucalypti in Northeast China
Source: Microorganisms. 2025 Jun 13;13(6):1376. doi: 10.3390/microorganisms13061376 (PMC12195282; doi:10.3390/microorganisms13061376)
Supplement: Supplementary file 1 [file microorganisms-13-01376-s001.zip › Table S4.pdf]

Table S4: Gene counts and functional descriptions of COG categories in the *P. eucalypti* GY78-10 genome.

Classification	Replicon	Chr	Plasmid 1	Plasmid 2	Plasmid 3	Total
Cellular Processes and Signaling	**D**	70	5	5	7	87
	**M**	293	12	13	7	325
	**N**	75	8	4	2	89
	**O**	194	15	7	5	221
	**T**	221	36	21	6	284
	**U**	103	8	3	4	118
	**V**	107	11	10	2	130
	**W**	30	5	2	2	39
	**Y**	0	0	0	0	0
	**Z**	2	0	0	0	2
Information Storage and Processing	**A**	1	0	0	0	1
	**B**	0	0	0	0	0
	**J**	276	12	6	0	294
	**K**	337	76	22	5	440
	**L**	159	3	3	12	177
	**X**	67	10	5	6	88
Metabolism	**C**	185	25	3	4	217
	**E**	378	51	4	3	436
	**F**	110	6	0	2	118
	**G**	372	61	13	9	455
	**H**	198	23	6	0	227
	**I**	152	20	4	5	181
	**P**	237	38	8	5	288
	**Q**	46	17	3	4	70
Poorly Characterized	**R**	297	61	16	8	382
	**S**	187	39	10	7	243

Abbreviation	Discription
D	Cell cycle control, cell division, chromosome partitioning
M	Cell wall/membrane/envelope biogenesis
N	Cell motility
O	Posttranslational modification, protein turnover, chaperones
T	Signal transduction mechanisms
U	Intracellular trafficking, secretion, and vesicular transport
V	Defense mechanisms
W	Extracellular structures
Y	Nuclear structure
Z	Cytoskeleton

A	RNA processing and modification
B	Chromatin structure and dynamics
J	Translation, ribosomal structure and biogenesis
K	Transcription
L	Replication, recombination and repair
X	Mobilome: prophages, transposons.
C	Energy production and conversion
E	Amino acid transport and metabolism
F	Nucleotide transport and metabolism
G	Carbohydrate transport and metabolism
H	Coenzyme transport and metabolism
I	Lipid transport and metabolism
P	Inorganic ion transport and metabolism
Q	Secondary metabolites biosynthesis, transport and catabolism
R	General function prediction only
S	Function unknown
Unclassified	
