# Supplementary material for: An Emerging Bacterial Leaf Disease in Rice Caused by Pantoea ananatis and Pantoea eucalypti in Northeast China
Source: Microorganisms. 2025 Jun 13;13(6):1376. doi: 10.3390/microorganisms13061376 (PMC12195282; doi:10.3390/microorganisms13061376)
Supplement: Supplementary file 1 [file microorganisms-13-01376-s001.zip › Table S5.pdf]

Table S5: Genomic identification of secretion systems, pathogenicity-related genes, genomic islands, and secondary metabolite biosynthetic gene clusters

1. **Identification of pathogenicity-related genes**

Protein_ID	PHI_ID	Pathogen_Species	E-value	identity (%)
GE000910	PHI:12256	Pantoea_stewartii	3.20E-120	100.00
GE002559	PHI:7161	Erwinia_amylovora	3.39E-38	100.00
GE003057	PHI:2685	Salmonella_enterica	4.65E-177	99.58
GE003028	PHI:6616	Klebsiella_pneumoniae	1.62E-158	99.52
GE002951	PHI:10577	Pectobacterium_carotovorum	7.60E-68	98.98
GE001736	PHI:7262	Salmonella_enterica	4.12E-45	98.55
GE002299	PHI:9311	Erwinia_amylovora	1.08E-55	96.47
GE002176	PHI:2499_PHI:4479	Erwinia_amylovora	5.83E-151	96.28
GE002575	PHI:3336	Erwinia_amylovora	0	96.06
GE001294	PHI:124344	Klebsiella_pneumoniae	2.77E-66	95.92
GE003261	PHI:9309	Erwinia_amylovora	0	95.63
GE003567	PHI:3074	Edwardsiella_tarda	1.60E-57	95.56
GE002416	PHI:2680	Salmonella_enterica	2.89E-136	95.29
GE001833	PHI:6361	Pantoea_ananatis	0	95.25
GE000571	PHI:7160	Erwinia_amylovora	0	95.03
GE003231	PHI:3092	Pantoea_stewartii	0	94.75
GE000382	PHI:6508	Salmonella_enterica	3.19E-107	94.70
GE002911	PHI:9566	Pantoea_ananatis	7.23E-70	94.23
GE000683	PHI:7263	Salmonella_enterica	4.62E-44	94.20
GE003234	PHI:9316	Erwinia_amylovora	0	94.06
GE002365	PHI:9306	Erwinia_amylovora	0	93.65
GE000924	PHI:2673	Salmonella_enterica	3.05E-63	93.62
GE003242	PHI:2695	Pectobacterium_atrosepticum	9.42E-72	93.33
GE001899	PHI:12255	Pantoea_stewartii	8.92E-148	92.89
GE003265	PHI:8512_PHI:8688_PHI:123999	Citrobacter_rodentium	3.03E-145	92.67
GE002217	PHI:9317	Erwinia_amylovora	0	92.48
GE001314	PHI:10106	Salmonella_enterica	6.75E-47	92.31
GE003315	PHI:123920	Escherichia_coli	0	92.17
GE002386	PHI:12253	Pantoea_stewartii	3.01E-111	92.12
GE002410	PHI:3724	Salmonella_enterica	5.01E-150	92.04
GE000272	PHI:6532_PHI:124163	Escherichia_coli	5.20E-166	92.02
GE003239	PHI:9325	Erwinia_amylovora	0	91.92
GE002086	PHI:2652	Klebsiella_pneumoniae	1.27E-127	91.71
GE000712	PHI:9327	Erwinia_amylovora	0	91.70

GE001651	PHI:9321	Erwinia_amylovora	0	91.67
GE003486	PHI:2644	Salmonella_enterica	1.29E-71	91.67
GE001152	PHI:7099_PHI:7408	Erwinia_amylovora	0	91.58
GE000354	PHI:3135	Yersinia_pseudotuberculosis	7.80E-160	91.34
GE000569	PHI:3040	Salmonella_enterica	1.29E-143	91.30
GE001571	PHI:4876_PHI:7248	Escherichia_coli	5.72E-171	91.20
GE000720	PHI:11509	Salmonella_enterica	6.41E-98	91.10
GE003621	PHI:3091	Pantoea_stewartii	1.71E-103	90.91
GE000529	PHI:123534	Salmonella_enterica	8.41E-158	90.83
GE000741	PHI:7964	Escherichia_coli	0	90.80
GE003216	PHI:9318	Erwinia_amylovora	0	90.66
GE003509	PHI:2479	Pectobacterium_carotovorum	0	90.54
GE003524	PHI:9326	Erwinia_amylovora	0	90.52
GE001315	PHI:3134	Yersinia_pseudotuberculosis	0	90.43
GE001309	PHI:6894	Salmonella_enterica	3.00E-170	90.32
GE001421	PHI:6449	Klebsiella_pneumoniae	0	90.12
GE000904	PHI:8569	Dickeya_dadantii	0	90.11
GE001824	PHI:8732_PHI:10049	Salmonella_enterica	8.07E-83	89.92
GE002111	PHI:11383	Salmonella_enterica	2.59E-130	89.84
GE000124	PHI:2459	Erwinia_amylovora	0	89.80
GE000123	PHI:3878	Salmonella_enterica	0	89.77
GE003325	PHI:7742	Salmonella_enterica	0	89.77
GE003056	PHI:2686	Salmonella_enterica	0	89.73
GE000960	PHI:3676_PHI:3682	Erwinia_amylovora	1.20E-33	89.09
GE001742	PHI:124002	Citrobacter_rodentium	0	89.08
GE002300	PHI:9312	Erwinia_amylovora	0	88.70
GE003238	PHI:9322	Erwinia_amylovora	0	88.56
GE003475	PHI:124230	Salmonella_enterica	0	88.55
GE000025	PHI:7081_PHI:7443	Erwinia_amylovora	0	88.50
GE003324	PHI:11030	Yersinia_pestis	0	88.47
GE003571	PHI:9313	Erwinia_amylovora	0	88.35
GE000089	PHI:2906_PHI:3673_PHI:3679	Erwinia_amylovora	0	88.34
GE002048	PHI:5109	Salmonella_enterica	0	88.29
GE000742	PHI:7965	Escherichia_coli	1.80E-162	88.24
GE003030	PHI:3126_PHI:9305	Erwinia_amylovora	0	88.12
GE002208	PHI:123168	Salmonella_enterica	0	87.94
GE000366	PHI:9152	Escherichia_coli	0	87.88

GE003440	PHI:11376	Salmonella_enterica	2.55E-136	87.86
GE003747	PHI:10046	Salmonella_enterica	0	87.85
GE002924	PHI:10537	Citrobacter_rodentium	1.16E-99	87.82
GE003371	PHI:123166	Salmonella_enterica	9.06E-157	87.82
GE000631	PHI:11418	Salmonella_enterica	2.51E-109	87.81
GE003357	PHI:9436	Salmonella_enterica	6.69E-172	87.78
GE000321	PHI:2672	Salmonella_enterica	0	87.69
GE001151	PHI:7098_PHI:7409	Erwinia_amylovora	7.53E-110	87.50
GE000913	PHI:6447	Klebsiella_pneumoniae	0	87.47
GE000704	PHI:124243	Escherichia_coli	0	87.45
GE000316	PHI:8964	Klebsiella_pneumoniae	0	87.26
GE000894	PHI:10536	Citrobacter_rodentium	5.84E-158	87.19
GE002400	PHI:9320	Erwinia_amylovora	0	87.11
GE002591	PHI:9323	Erwinia_amylovora	0	87.04
GE002906	PHI:2625_PHI:9416	Salmonella_enterica	0	87.04
GE003366	PHI:9308	Erwinia_amylovora	0	87.03
GE003581	PHI:9310	Erwinia_amylovora	0	86.86
GE001415	PHI:10048	Salmonella_enterica	0	86.83
GE003423	PHI:10919	Erwinia_amylovora	0	86.73
GE000596	PHI:2469	Erwinia_amylovora	0	86.64
GE002497	PHI:3675_PHI:3681	Erwinia_amylovora	9.74E-67	86.61
GE000756	PHI:10905	Citrobacter_rodentium	0	86.54
GE000294	PHI:8577	Dickeya_dadantii	0	86.51
GE003059	PHI:7131	Salmonella_enterica	9.31E-97	86.45
GE000088	PHI:3674_PHI:3680	Erwinia_amylovora	1.39E-57	86.32
GE003525	PHI:9315	Erwinia_amylovora	0	86.30
GE001607	PHI:7657	Salmonella_enterica	3.84E-130	86.18
GE001308	PHI:6893_PHI:10099	Salmonella_enterica	0	86.15
GE003266	PHI:8513_PHI:8689_PHI:124000	Citrobacter_rodentium	0	86.12
GE001821	PHI:8731	Salmonella_enterica	0	86.06
GE002355	PHI:11759	Escherichia_coli	0	86.05
GE002570	PHI:10513	Escherichia_coli	0	85.83
GE002428	PHI:4218	Escherichia_coli	1.53E-106	85.63
GE004005	PHI:10084	Salmonella_enterica	1.24E-176	85.61
GE000607	PHI:6472	Escherichia_coli	0	85.58
GE001980	PHI:12254	Pantoea_stewartii	0	85.53
GE000402	PHI:9334	Citrobacter_rodentium	1.43E-172	85.39
GE001130	PHI:7528	Klebsiella_pneumoniae	0	85.31

GE003629	PHI:9229	Escherichia_coli	0	85.27
GE000997	PHI:560	Salmonella_enterica	3.88E-139	85.25
GE001825	PHI:6461	Salmonella_enterica	0	85.10
GE003631	PHI:9227	Escherichia_coli	0	85.10
GE001126	PHI:7533	Klebsiella_pneumoniae	0	85.08
GE002493	PHI:10595	Pectobacterium_carotovorum	0	85.06
GE003297	PHI:7662	Salmonella_enterica	8.99E-139	84.98
GE001834	PHI:8572	Dickeya_dadantii	8.24E-119	84.97
GE001790	PHI:11149	Pectobacterium_atrosepticum	9.66E-133	84.91
GE000317	PHI:8862	Salmonella_enterica	0	84.77
GE002934	PHI:124123	Yersinia_enterocolitica	0	84.62
GE001665	PHI:123208	Salmonella_enterica	0	84.57
GE001740	PHI:4175	Dickeya_solani	5.29E-167	84.41
GE000588	PHI:11219	Salmonella_enterica	1.99E-41	84.29
GE000318	PHI:8962	Klebsiella_pneumoniae	0	83.94
GE001100	PHI:9465	Salmonella_enterica	1.71E-137	83.78
GE002905	PHI:12252	Pantoea_stewartii	3.17E-91	83.67
GE000921	PHI:9459	Salmonella_enterica	0	83.57
GE000087	PHI:3133	Yersinia_pseudotuberculosis	3.78E-96	83.54
GE000109	PHI:7130	Salmonella_enterica	5.18E-99	83.54
GE002394	PHI:2962	Edwardsiella_ictaluri	0	83.45
GE002598	PHI:9161	Salmonella_enterica	0	83.33
GE003034	PHI:11417	Salmonella_enterica	2.98E-108	83.16
GE002479	PHI:8968	Klebsiella_pneumoniae	0	83.08
GE002571	PHI:10512	Escherichia_coli	0	83.00
GE001167	PHI:7106_PHI:7393	Erwinia_amylovora	0	82.99
GE002094	PHI:9198	Escherichia_coli	0	82.84
GE001754	PHI:7520	Klebsiella_pneumoniae	0	82.68
GE002016	PHI:11381	Salmonella_enterica	0	82.66
GE003241	PHI:8570	Dickeya_dadantii	0	82.60
GE001830	PHI:6536	Escherichia_coli	1.41E-95	82.32
GE003083	PHI:10051	Salmonella_enterica	0	82.14
GE001154	PHI:3408	Pantoea_ananatis	6.97E-93	81.88
GE002193	PHI:8937	Pseudomonas_aeruginosa	1.14E-107	81.82
GE001743	PHI:9896	Escherichia_coli	0	81.75
GE002323	PHI:12047	Salmonella_enterica	0	81.56
GE000068	PHI:6420	Salmonella_enterica	9.36E-117	81.38
GE001789	PHI:8653	Escherichia_coli	0	81.38
GE000130	PHI:11411	Klebsiella_pneumoniae	0	81.27
GE001799	PHI:11006	Escherichia_coli	2.20E-149	81.15
GE001606	PHI:7658	Salmonella_enterica	6.05E-37	81.08
GE000315	PHI:8965	Klebsiella_pneumoniae	2.69E-123	81.00
GE001679	PHI:124019	Salmonella_enterica	0	80.80

GE000367	PHI:9153	Escherichia_coli	6.71E-178	80.70
GE003072	PHI:123652	Escherichia_coli	0	80.64
GE002134	PHI:11609	Salmonella_enterica	2.89E-175	80.42
GE004006	PHI:10083	Salmonella_enterica	3.31E-155	80.39
GE000029	PHI:7108_PHI:7439	Erwinia_amylovora	1.95E-101	80.37
GE003632	PHI:124242	Escherichia_coli	0	80.33
GE000347	PHI:6362	Pantoea_ananatis	4.37E-87	80.27
GE003535	PHI:10983	Escherichia_coli	1.11E-150	80.24
GE002483	PHI:5407_PHI:7646	Escherichia_coli	6.20E-101	80.12
GE000770	PHI:6269	Escherichia_coli	0	80.11
GE002053	PHI:9324	Erwinia_amylovora	1.65E-143	80.00
GE002792	PHI:124003	Citrobacter_rodentium	3.86E-119	79.91
GE003011	PHI:4584	Cronobacter_universalis	5.73E-136	79.62
GE002686	PHI:9150	Escherichia_coli	0	79.61
GE001161	PHI:7399	Erwinia_amylovora	6.51E-157	79.55
GE000739	PHI:7962_PHI:123646	Escherichia_coli	1.37E-72	79.53
GE002796	PHI:4186	Yersinia_pestis	2.00E-44	79.52
GE002568	PHI:10515	Escherichia_coli	1.35E-134	79.46
GE001823	PHI:10050	Salmonella_enterica	5.54E-111	79.33
GE001798	PHI:11007	Escherichia_coli	4.49E-147	79.28
GE004174	PHI:7951	Klebsiella_pneumoniae	0	79.06
GE001411	PHI:4631	Escherichia_coli	0	79.04
GE002555	PHI:3731	Escherichia_coli	2.11E-104	78.95
GE000312	PHI:123649	Escherichia_coli	0	78.80
GE002699	PHI:6363	Pantoea_ananatis	0	78.79
GE000301	PHI:10573	Pectobacterium_carotovorum	1.83E-163	78.68
GE002923	PHI:2959	Edwardsiella_ictaluri	1.00E-179	78.53
GE001835	PHI:12271_PHI:12272_PHI:12275_PHI:12276	Serratia_marcescens	1.23E-63	78.45
GE001354	PHI:11380	Salmonella_enterica	0	78.40
GE001043	PHI:9307	Erwinia_amylovora	0	78.22
GE000278	PHI:11408	Klebsiella_pneumoniae	2.60E-145	78.21
GE000964	PHI:4160	Klebsiella_pneumoniae	0	78.09
GE003263	PHI:10387	Salmonella_enterica	3.94E-19	78.00
GE000790	PHI:10987	Escherichia_coli	0	77.98
GE002577	PHI:6187	Salmonella_enterica	1.22E-118	77.89
GE001708	PHI:11407	Klebsiella_pneumoniae	3.14E-135	77.64
GE000397	PHI:8690	Citrobacter_rodentium	0	77.59
GE001062	PHI:6360	Pantoea_ananatis	0	77.56
GE000707	PHI:7671	Yersinia_enterocolitica	2.71E-86	77.42

GE002489	PHI:5571_PHI:6366	Salmonella_enterica	0	77.39
GE002987	PHI:11120	Bordetella_pertussis	2.01E-64	77.24
GE001281	PHI:10587	Pectobacterium_carotovorum	4.02E-147	77.08
GE002665	PHI:2961	Edwardsiella_ictaluri	0	76.96
GE001336	PHI:11987	Serratia_marcescens	5.43E-78	76.92
GE001676	PHI:6369	Escherichia_coli	2.26E-64	76.81
GE002710	PHI:9151	Escherichia_coli	0	76.62
GE001849	PHI:10991	Escherichia_coli	0	76.60
GE002592	PHI:8574	Dickeya_dadantii	0	76.59
GE000083	PHI:124164	Escherichia_coli	0	76.45
GE004173	PHI:7950	Klebsiella_pneumoniae	0	76.40
GE000055	PHI:7082_PHI:7418	Erwinia_amylovora	1.96E-78	76.39
GE004007	PHI:10082	Salmonella_enterica	2.50E-178	76.32
GE001831	PHI:6535	Escherichia_coli	0	76.19
GE001165	PHI:7101_PHI:7395	Erwinia_amylovora	0	76.12
GE002942	PHI:124212	Salmonella_enterica	0	76.05
GE000570	PHI:9833	Xanthomonas_campestris	0	75.96
GE001147	PHI:7103_PHI:7413	Erwinia_amylovora	0	75.73
GE001148	PHI:7090_PHI:7412	Erwinia_amylovora	0	75.62
GE001753	PHI:7521	Klebsiella_pneumoniae	1.41E-104	75.52
GE000982	PHI:10387	Salmonella_enterica	4.23E-21	75.51
GE002731	PHI:10387	Salmonella_enterica	7.93E-22	75.51
GE000903	PHI:8568	Dickeya_dadantii	5.02E-57	75.47
GE002892	PHI:7020	Escherichia_coli	0	75.43
GE001096	PHI:5572	Salmonella_enterica	2.92E-148	75.37
GE003003	PHI:10282	Staphylococcus_aureus	0	75.32
GE003461	PHI:124154	Dickeya_solani	0	75.25
GE002351	PHI:8652	Escherichia_coli	3.31E-117	74.76
GE002668	PHI:7028	Escherichia_coli	0	74.75
GE001626	PHI:5503	Cronobacter_turicensis	4.21E-170	74.74
GE002791	PHI:6553	Salmonella_enterica	0	74.59
GE003549	PHI:10282	Staphylococcus_aureus	0	74.56
GE003751	PHI:123638	Salmonella_enterica	1.77E-133	74.48
GE001848	PHI:10992	Escherichia_coli	0	74.47
GE001037	PHI:3166	Pseudomonas_aeruginosa	1.19E-102	74.35
GE001600	PHI:4563	Yersinia_pestis	0	74.28
GE001422	PHI:6448	Klebsiella_pneumoniae	0	74.27
GE001053	PHI:124366	Salmonella_enterica	4.64E-73	74.27
GE002080	PHI:2471	Erwinia_amylovora	0	74.26

GE000740	PHI:7963	Escherichia_coli	4.48E-50	73.91
GE003488	PHI:4074	Serratia_marcescens	1.90E-177	73.90
GE000286	PHI:3267	Escherichia_coli	7.58E-164	73.81
GE001164	PHI:7100_PHI:7396	Erwinia_amylovora	3.82E-99	73.77
GE001826	PHI:11916	Salmonella_enterica	4.32E-159	73.74
GE002572	PHI:123379	Salmonella_enterica	0	73.42
GE001311	PHI:10102	Salmonella_enterica	0	73.40
GE001757	PHI:8967	Klebsiella_pneumoniae	3.20E-51	73.33
GE000030	PHI:7094_PHI:7438	Erwinia_amylovora	0	73.33
GE000854	PHI:6250_PHI:124233	Escherichia_coli	2.23E-85	73.26
GE002201	PHI:5089	Yersinia_pestis	1.46E-168	73.20
GE003482	PHI:11419	Salmonella_enterica	7.26E-48	73.12
GE002852	PHI:11405	Klebsiella_pneumoniae	7.26E-112	73.06
GE001578	PHI:9601	Escherichia_coli	3.49E-117	73.03
GE002132	PHI:11344	Escherichia_coli	3.74E-160	72.91
GE002067	PHI:7634	Pseudomonas_aeruginosa	7.93E-161	72.82
GE000282	PHI:9230	Escherichia_coli	5.55E-94	72.73
GE001625	PHI:5504	Cronobacter_turicensis	0	72.59
GE001031	PHI:5564_PHI:8654	Dickeya_dadantii	0	72.59
GE000074	PHI:8575	Dickeya_dadantii	0	72.49
GE002562	PHI:10380	Acinetobacter_baumannii	0	72.46
GE000263	PHI:10580	Pectobacterium_carotovorum	3.82E-117	72.44
GE000530	PHI:9434	Salmonella_enterica	0	72.26
GE002186	PHI:10993	Escherichia_coli	0	72.23
GE002648	PHI:8581	Dickeya_dadantii	0	72.14
GE002623	PHI:8729	Salmonella_enterica	0	72.12
GE004063	PHI:3751	Salmonella_enterica	1.33E-51	72.12
GE001150	PHI:3407	Pantoea_ananatis	0	72.06
GE003762	PHI:7263	Salmonella_enterica	2.18E-32	72.06
GE003763	PHI:7263	Salmonella_enterica	2.18E-32	72.06
GE000024	PHI:7080_PHI:7444	Erwinia_amylovora	8.03E-84	71.88
GE001394	PHI:4179	Dickeya_solani	1.42E-35	71.83
GE001747	PHI:11187	Yersinia_pseudotuberculosis	0	71.63
GE002207	PHI:9843	Vibrio_cholerae	0	71.61
GE001797	PHI:11005	Escherichia_coli	8.23E-153	71.53
GE001169	PHI:7092	Erwinia_amylovora	0	71.52
GE000938	PHI:11001	Escherichia_coli	8.03E-100	71.43
GE003346	PHI:6882	Salmonella_enterica	9.76E-111	71.23
GE002573	PHI:124167	Escherichia_coli	5.09E-88	71.22

GE001680	PHI:124165	Escherichia_coli	9.40E-175	71.22
GE000951	PHI:11451	Erwinia_amylovora	0	71.05
GE003630	PHI:9228	Escherichia_coli	4.85E-49	71.00
GE001839	PHI:4497	Salmonella_enterica	1.64E-111	70.91
GE002145	PHI:11093	Escherichia_coli	4.63E-93	70.79
GE000485	PHI:7263	Salmonella_enterica	1.36E-31	70.59
GE001796	PHI:4185	Yersinia_pestis	0	70.55
GE000589	PHI:11220	Salmonella_enterica	3.71E-59	70.54
GE004172	PHI:7949	Klebsiella_pneumoniae	0	70.48
GE000599	PHI:8933	Pectobacterium_atrosepticum	1.15E-14	70.46
GE001146	PHI:7087_PHI:7414	Erwinia_amylovora	0	70.40
GE001717	PHI:4108	Vibrio_cholerae	0	70.34
GE002779	PHI:10978	Escherichia_coli	0	70.32
GE003569	PHI:3942	Xanthomonas_campestris	0	70.26
GE002257	PHI:7534	Klebsiella_pneumoniae	2.60E-61	70.23
GE003556	PHI:7860	Acinetobacter_baumannii	0	70.02
GE003533	PHI:8757	Salmonella_enterica	2.06E-32	70.00
GE000304	PHI:10312	Escherichia_coli	0	69.94
GE001979	PHI:10578	Pectobacterium_carotovorum	5.93E-162	69.91
GE002155	PHI:7469	Pectobacterium_carotovorum	2.32E-44	69.89
GE003333	PHI:8691	Citrobacter_rodentium	7.04E-107	69.86
GE003327	PHI:123267	Pseudomonas_aeruginosa	0	69.74
GE000799	PHI:123209	Salmonella_enterica	0	69.73
GE001900	PHI:6982	Salmonella_enterica	0	69.60
GE002797	PHI:3147	Glaesserella_parasuis	0	69.59
GE000285	PHI:3266	Escherichia_coli	3.98E-171	69.37
GE001714	PHI:123440	Xanthomonas_oryzae	4.63E-136	69.26
GE001744	PHI:11739	Dickeya_dadantii	3.92E-113	69.08
GE003223	PHI:8567	Dickeya_dadantii	1.33E-136	69.00
GE003281	PHI:9594	Salmonella_enterica	2.57E-163	68.99
GE003474	PHI:8644_PHI:8642	Erwinia_amylovora	0	68.93
GE002576	PHI:9369	Yersinia_pestis	1.55E-139	68.81
GE000941	PHI:7023	Escherichia_coli	0	68.72
GE004004	PHI:10085	Salmonella_enterica	1.18E-135	68.66
GE002463	PHI:7573	Yersinia_pestis	0	68.38
GE002175	PHI:2501	Erwinia_amylovora	0	68.33
GE003174	PHI:124124	Yersinia_enterocolitica	0	68.31
GE000861	PHI:6097	Klebsiella_pneumoniae	3.38E-53	68.15
GE002264	PHI:3408	Pantoea_ananatis	5.50E-74	67.93
GE003221	PHI:7572	Klebsiella_pneumoniae	0	67.85
GE002586	PHI:1138	Xanthomonas_oryzae	2.45E-151	67.68
GE001166	PHI:7084_PHI:73	Erwinia_amylovora	1.30E-162	67.67

	94			
GE002131	PHI:11343	Escherichia_coli	4.74E-125	67.59
GE002312	PHI:8728	Salmonella_enterica	3.94E-136	67.50
GE002399	PHI:12149	Escherichia_coli	0	67.30
GE001788	PHI:124001	Citrobacter_rodentium	3.40E-87	67.19
GE001526	PHI:9586	Xanthomonas_oryzae	6.33E-35	67.05
GE002097	PHI:123641	Escherichia_coli	0	66.96
GE001832	PHI:6534	Escherichia_coli	5.58E-153	66.89
GE002919	PHI:6274	Pseudomonas_aeruginosa	1.56E-88	66.85
GE002168	PHI:4161	Klebsiella_pneumoniae	5.10E-175	66.76
GE002177	PHI:4480	Erwinia_amylovora	0	66.70
GE002714	PHI:7908	Yersinia_pseudotuberculosis	3.01E-139	66.67
GE002829	PHI:10946	Pseudomonas_aeruginosa	5.13E-19	66.67
GE004204	PHI:10630	Burkholderia_glumae	1.89E-14	66.67
GE004234	PHI:10630	Burkholderia_glumae	1.89E-14	66.67
GE004296	PHI:10946	Pseudomonas_aeruginosa	8.02E-20	66.67
GE003370	PHI:3412	Xanthomonas_citri	1.00E-130	66.53
GE001575	PHI:3780	Salmonella_enterica	2.93E-138	66.35
GE001029	PHI:123210	Salmonella_enterica	0	66.26
GE002965	PHI:2694	Pectobacterium_atrosepticum	0	66.20
GE003208	PHI:7699	Pseudomonas_syringae	3.10E-103	66.18
GE002195	PHI:11247	Pseudomonas_aeruginosa	0	66.08
GE003039	PHI:123790	Francisella_tularensis	3.44E-102	66.06
GE002192	PHI:8938	Pseudomonas_aeruginosa	4.26E-60	66.06
GE001565	PHI:6985	Salmonella_enterica	0	65.88
GE001174	PHI:7092	Erwinia_amylovora	0	65.81
GE002383	PHI:3761	Salmonella_enterica	5.13E-116	65.75
GE002379	PHI:6438	Pseudomonas_aeruginosa	4.23E-68	65.73
GE000572	PHI:8676	Xanthomonas_citri	9.94E-37	65.56
GE001898	PHI:12269_PHI:12270_PHI:12273_PHI:12274	Serratia_marcescens	4.74E-103	65.48
GE000284	PHI:10485	Pseudomonas_aeruginosa	2.22E-173	65.37
GE002596	PHI:123626	Xanthomonas_albilineans	0	65.31
GE003471	PHI:4694	Salmonella_enterica	0	65.31
GE001756	PHI:8966	Klebsiella_pneumoniae	3.10E-47	65.25
GE004199	PHI:6097	Klebsiella_pneumoniae	2.28E-48	65.19
GE004229	PHI:6097	Klebsiella_pneumoniae	2.28E-48	65.19
GE002636	PHI:7474	Pseudomonas_aeruginosa	6.13E-76	64.97
GE003732	PHI:123650	Escherichia_coli	3.07E-138	64.97
GE001310	PHI:10101	Salmonella_enterica	0	64.95
GE001822	PHI:11174	Escherichia_coli	1.13E-173	64.72
GE000507	PHI:6268_PHI:9897	Escherichia_coli	0	64.66

GE001024	PHI:5272	Ralstonia_solanacearum	1.83E-112	64.61
GE001331	PHI:9809_PHI:11379	Salmonella_enterica	8.66E-79	64.54
GE002887	PHI:10714	Edwardsiella_tarda	0	64.51
GE002092	PHI:3947	Xanthomonas_campestris	7.76E-144	64.44
GE003334	PHI:8692	Citrobacter_rodentium	3.89E-167	64.33
GE002944	PHI:124161	Ralstonia_solanacearum	5.92E-57	64.29
GE001471	PHI:4717	Pseudomonas_aeruginosa	0	64.27
GE001072	PHI:5271	Ralstonia_solanacearum	5.21E-106	64.08
GE003844	PHI:7956	Klebsiella_pneumoniae	9.28E-89	64.02
GE001637	PHI:8764	Pseudomonas_aeruginosa	6.34E-158	63.98
GE000767	PHI:8884	Ralstonia_solanacearum	3.13E-170	63.92
GE004329	PHI:3165	Pseudomonas_cichorii	2.11E-99	63.90
GE000829	PHI:11409	Klebsiella_pneumoniae	3.18E-117	63.90
GE001312	PHI:10103	Salmonella_enterica	8.28E-62	63.77
GE004175	PHI:7952	Klebsiella_pneumoniae	2.95E-139	63.73
GE000369	PHI:10985	Escherichia_coli	0	63.69
GE003476	PHI:4189	Yersinia_pestis	0	63.66
GE002128	PHI:3277	Aspergillus_fumigatus	3.52E-179	63.61
GE001727	PHI:9496	Salmonella_enterica	1.49E-77	63.59
GE000391	PHI:3950	Xanthomonas_campestris	0	63.53
GE002171	PHI:3710	Pectobacterium_carotovorum	0	63.51
GE001035	PHI:7947	Klebsiella_pneumoniae	2.25E-149	63.43
GE002070	PHI:9264	Haemophilus_influenzae	7.81E-160	63.21
GE000057	PHI:7079_PHI:7417	Erwinia_amylovora	0	63.18
GE003602	PHI:7661	Salmonella_enterica	2.27E-26	63.16
GE001168	PHI:7392	Erwinia_amylovora	0	63.07
GE000065	PHI:3085	Porphyromonas_gingivalis	0	63.03
GE001051	PHI:124362	Salmonella_enterica	7.01E-37	63.00
GE001244	PHI:7105	Erwinia_amylovora	0	62.96
GE002382	PHI:609_PHI:616	Salmonella_enterica	1.69E-119	62.95
GE001866	PHI:6733	Escherichia_coli	0	62.79
GE001099	PHI:3725	Salmonella_enterica	0	62.71
GE001020	PHI:9197	Escherichia_coli	0	62.50
GE002716	PHI:7906	Yersinia_pseudotuberculosis	1.54E-154	62.46
GE000001	PHI:11544	Brucella_melitensis	1.30E-164	62.38
GE002235	PHI:123734_PHI:123729	Xanthomonas_campestris	1.33E-177	62.34
GE000061	PHI:123643	Escherichia_coli	0	62.21
GE001160	PHI:7095_PHI:7400	Erwinia_amylovora	0	62.15
GE001064	PHI:3723	Salmonella_enterica	0	62.12
GE000052	PHI:7083_PHI:74	Erwinia_amylovora	0	61.80

	30			
GE003176	PHI:4712	Pseudomonas_aeruginosa	0	61.76
GE001266	PHI:3126_PHI:9305	Erwinia_amylovora	0	61.71
GE001135	PHI:10199	Escherichia_coli	0	61.67
GE001149	PHI:7411	Erwinia_amylovora	9.09E-107	61.60
GE000961	PHI:123733_PHI:123728	Xanthomonas_campestris	6.86E-78	61.54
GE003377	PHI:9129	Brucella_abortus	9.89E-135	61.39
GE002251	PHI:123645	Escherichia_coli	6.77E-48	61.35
GE000357	PHI:6520	Legionella_pneumophila	0	61.26
GE001890	PHI:6463	Salmonella_enterica	9.19E-59	61.22
GE003451	PHI:10163	Burkholderia_thailandensis	9.64E-57	61.19
GE002395	PHI:123380	Salmonella_enterica	9.22E-179	61.17
GE001889	PHI:6995	Pseudomonas_aeruginosa	3.52E-180	61.16
GE002600	PHI:562	Salmonella_enterica	0	61.09
GE001163	PHI:7397	Erwinia_amylovora	1.00E-109	61.03
GE002696	PHI:9281	Pseudomonas_aeruginosa	1.48E-140	61.02
GE000102	PHI:7630	Pseudomonas_aeruginosa	0	60.86
GE004176	PHI:7953	Klebsiella_pneumoniae	0	60.74
GE001827	PHI:6981	Salmonella_enterica	0	60.71
GE001242	PHI:7096	Erwinia_amylovora	1.48E-147	60.65
GE000155	PHI:2634	Staphylococcus_aureus	7.14E-151	60.61
GE004192	PHI:6941	Pseudomonas_aeruginosa	0	60.47
GE004222	PHI:6941	Pseudomonas_aeruginosa	0	60.47
GE002179	PHI:824_PHI:6838	Burkholderia_glumae	0	60.31
GE002761	PHI:7225	Salmonella_enterica	0	60.21
GE000158	PHI:2634	Staphylococcus_aureus	1.84E-149	60.18
GE000349	PHI:124179	Pseudomonas_aeruginosa	6.53E-74	60.11
GE001623	PHI:7577	Salmonella_enterica	0	60.09
GE000144	PHI:9198	Escherichia_coli	0	60.08
GE001541	PHI:11882	Salmonella_enterica	2.75E-32	60.00
GE001542	PHI:11882	Salmonella_enterica	2.67E-30	60.00
GE001828	PHI:6981	Salmonella_enterica	0	59.96
GE001860	PHI:3922	Xenorhabdus_nematophila	1.30E-75	59.88
GE000716	PHI:5472	Magnaporthe_oryzae	2.95E-118	59.85
GE003273	PHI:5573	Salmonella_enterica	8.36E-165	59.77
GE001245	PHI:7104	Erwinia_amylovora	2.54E-162	59.65
GE002202	PHI:123722	Paracidovorax_citrulli	0	59.60
GE003031	PHI:3941	Xanthomonas_campestris	8.62E-83	59.38
GE004201	PHI:6369	Escherichia_coli	2.19E-41	59.26
GE004231	PHI:6369	Escherichia_coli	2.19E-41	59.26
GE004205	PHI:7108_PHI:74	Erwinia_amylovora	9.26E-42	59.22

	39			
GE004235	PHI:7108_PHI:7439	Erwinia_amylovora	9.26E-42	59.22
GE003006	PHI:1599	Fusarium_graminearum	2.33E-43	59.20
GE000792	PHI:7748	Mycobacterium_tuberculosis	2.71E-48	59.12
GE000698	PHI:10313	Escherichia_coli	5.30E-69	59.04
GE003944	PHI:11791	Ralstonia_solanacearum	8.80E-171	59.04
GE001739	PHI:9521	Salmonella_enterica	3.72E-33	58.97
GE002071	PHI:124019	Salmonella_enterica	1.04E-122	58.92
GE000802	PHI:11622_PHI:11623	Pseudomonas_aeruginosa	1.27E-146	58.84
GE000339	PHI:5240	Acinetobacter_baumannii	0	58.77
GE003846	PHI:6531	Escherichia_coli	7.60E-157	58.54
GE002713	PHI:7909	Yersinia_pseudotuberculosis	2.10E-96	58.43
GE003426	PHI:11169	Escherichia_coli	8.36E-133	58.31
GE003612	PHI:2737	Xanthomonas_oryzae	1.66E-38	58.25
GE000492	PHI:10984	Escherichia_coli	0	58.23
GE000902	PHI:8639	Xanthomonas_oryzae	3.44E-26	58.21
GE002776	PHI:11405	Klebsiella_pneumoniae	3.25E-92	58.18
GE000687	PHI:6492	Acinetobacter_baumannii	4.72E-136	58.13
GE003211	PHI:123751	Salmonella_enterica	1.91E-128	58.11
GE004202	PHI:9575	Escherichia_coli	5.29E-136	58.10
GE004232	PHI:9575	Escherichia_coli	5.29E-136	58.10
GE004155	PHI:9026	Xanthomonas_oryzae	1.77E-93	58.08
GE000476	PHI:7023	Escherichia_coli	4.54E-145	57.87
GE000852	PHI:10828	Agrobacterium_tumefaciens	5.98E-61	57.79
GE001279	PHI:124083	Actinobacillus_pleuropneumoniae	3.36E-168	57.76
GE003534	PHI:8758	Salmonella_enterica	1.70E-60	57.73
GE001829	PHI:6981	Salmonella_enterica	0	57.71
GE003962	PHI:10988	Escherichia_coli	1.32E-63	57.65
GE003120	PHI:11870	Edwardsiella_tarda	0	57.63
GE002569	PHI:10514	Escherichia_coli	9.98E-90	57.51
GE001376	PHI:4981	Edwardsiella_tarda	8.31E-37	57.29
GE001605	PHI:7659	Salmonella_enterica	6.22E-45	57.14
GE004214	PHI:124113	Shigella_flexneri	0	56.99
GE002688	PHI:877	Magnaporthe_oryzae	6.56E-144	56.99
GE001516	PHI:6133	Salmonella_enterica	2.26E-159	56.81
GE001265	PHI:7023	Escherichia_coli	3.39E-139	56.79
GE001089	PHI:8939	Pseudomonas_aeruginosa	5.13E-168	56.68
GE002683	PHI:123788	Francisella_tularensis	0	56.66
GE000788	PHI:11576	Streptococcus_agalactiae	0	56.52
GE003347	PHI:3709	Pectobacterium_carotovorum	1.28E-167	56.50
GE002700	PHI:123731	Paracidovorax_citrulli	3.92E-76	56.49
GE003654	PHI:123859	Burkholderia_pseudomallei	2.27E-76	56.48

GE000597	PHI:11922	Pseudomonas_aeruginosa	1.84E-132	56.37
GE002952	PHI:11546	Brucella_melitensis	1.08E-137	56.31
GE000830	PHI:2458	Erwinia_amylovora	6.86E-51	56.30
GE001496	PHI:2651	Salmonella_enterica	8.23E-45	56.25
GE001886	PHI:123276	Proteus_mirabilis	0	56.22
GE002353	PHI:124153	Dickeya_solani	0	56.19
GE001719	PHI:6870	Vibrio_harveyi	1.08E-88	56.03
GE002315	PHI:123788	Francisella_tularensis	0	55.99
GE003042	PHI:4250	Burkholderia_glumae	4.12E-122	55.98
GE003658	PHI:2153_PHI:2356	Magnaporthe_oryzae	9.01E-62	55.98
GE003903	PHI:9334	Citrobacter_rodentium	6.55E-102	55.87
GE001145	PHI:7102_PHI:7415	Erwinia_amylovora	2.09E-49	55.86
GE000564	PHI:4147	Xanthomonas_citri	4.43E-120	55.78
GE004244	PHI:11923	Pseudomonas_aeruginosa	0	55.75
GE001301	PHI:8885	Ralstonia_solanacearum	3.54E-135	55.62
GE000389	PHI:10989	Escherichia_coli	1.22E-113	55.52
GE001246	PHI:7416	Erwinia_amylovora	2.34E-67	55.42
GE001836	PHI:9814	Ralstonia_solanacearum	0	55.34
GE001239	PHI:7392	Erwinia_amylovora	1.30E-175	55.33
GE003402	PHI:4102	Brucella_suis	5.59E-50	55.32
GE001355	PHI:124080	Streptococcus_suis	1.41E-65	55.31
GE002715	PHI:7907	Yersinia_pseudotuberculosis	3.45E-97	55.17
GE003502	PHI:9840	Vibrio_cholerae	0	55.09
GE003758	PHI:5059	Pseudomonas_aeruginosa	9.08E-175	54.97
GE003967	PHI:3211_PHI:3212	Pseudomonas_aeruginosa	1.37E-134	54.80
GE000031	PHI:8868	Escherichia_coli	0	54.77
GE002675	PHI:123789	Francisella_tularensis	8.47E-79	54.76
GE002388	PHI:3915	Pseudomonas_aeruginosa	1.44E-100	54.61
GE000728	PHI:10119	Salmonella_enterica	1.15E-129	54.60
GE003136	PHI:8643	Erwinia_amylovora	0	54.60
GE002500	PHI:8884	Ralstonia_solanacearum	1.79E-123	54.41
GE001259	PHI:7533	Klebsiella_pneumoniae	3.29E-113	54.27
GE001087	PHI:123476	Pseudomonas_aeruginosa	1.02E-95	54.26
GE003361	PHI:10773	Staphylococcus_aureus	0	54.10
GE003637	PHI:6979	Salmonella_enterica	0	54.07
GE002595	PHI:11234	Streptococcus_suis	4.78E-152	54.06
GE003365	PHI:8042	Acinetobacter_baumannii	2.06E-178	53.98
GE002584	PHI:10426	Magnaporthe_oryzae	1.34E-75	53.92
GE002580	PHI:4145	Xanthomonas_citri	3.85E-54	53.90
GE001226	PHI:4732	Francisella_tularensis	6.52E-126	53.82
GE000415	PHI:3785	Pseudomonas_aeruginosa	1.21E-89	53.82

GE000049	PHI:7091_PHI:7432	Erwinia_amylovora	0	53.70
GE000821	PHI:6981	Salmonella_enterica	4.82E-91	53.70
GE001856	PHI:8623	Neisseria_meningitidis	5.01E-89	53.69
GE002729	PHI:4137	Xanthomonas_oryzae	9.93E-56	53.66
GE001144	PHI:7416	Erwinia_amylovora	7.73E-73	53.65
GE000053	PHI:7085_PHI:7429	Erwinia_amylovora	3.80E-125	53.43
GE002003	PHI:6981	Salmonella_enterica	2.34E-104	53.43
GE001920	PHI:10919	Erwinia_amylovora	3.79E-157	53.40
GE000981	PHI:9134	Brucella_abortus	2.75E-63	53.27
GE000094	PHI:7067	Francisella_tularensis	2.66E-111	53.23
GE001604	PHI:7660	Salmonella_enterica	2.35E-21	53.23
GE002352	PHI:9450_PHI:9760_PHI:11314	Staphylococcus_aureus	4.13E-106	53.15
GE002417	PHI:8507	Coxiella_burnetii	0	53.10
GE002585	PHI:1139	Xanthomonas_oryzae	1.27E-143	52.88
GE003264	PHI:8515	Citrobacter_rodentium	8.24E-46	52.70
GE001966	PHI:11791	Ralstonia_solanacearum	2.37E-144	52.68
GE003655	PHI:5369	Salmonella_enterica	1.85E-37	52.68
GE001353	PHI:6980	Salmonella_enterica	0	52.63
GE002258	PHI:7534	Klebsiella_pneumoniae	2.96E-57	52.54
GE002049	PHI:9983	Xanthomonas_oryzae	2.24E-133	52.47
GE004033	PHI:6980	Salmonella_enterica	1.17E-81	52.44
GE000558	PHI:3259	Xanthomonas_campestris	2.64E-50	52.44
GE003620	PHI:7475	Klebsiella_pneumoniae	8.19E-36	52.43
GE003861	PHI:7882	Ralstonia_solanacearum	6.30E-98	52.41
GE003376	PHI:124300	Xanthomonas_citri	1.77E-168	52.30
GE000686	PHI:8757	Salmonella_enterica	3.79E-18	52.24
GE003319	PHI:9177	Salmonella_enterica	0	52.24
GE003450	PHI:10162	Burkholderia_thailandensis	5.88E-41	52.17
GE002051	PHI:7790	Xanthomonas_oryzae	6.29E-131	52.15
GE001978	PHI:3914	Parastagonospora_nodorum	5.66E-167	52.08
GE001073	PHI:8556	Pseudomonas_aeruginosa	4.09E-21	52.06
GE001659	PHI:6651	Klebsiella_pneumoniae	1.60E-59	52.05
GE003819	PHI:6985	Salmonella_enterica	2.03E-91	51.99
GE003287	PHI:7635	Pseudomonas_aeruginosa	2.92E-145	51.96
GE003139	PHI:8826	Burkholderia_cenocepacia	5.54E-112	51.91
GE003200	PHI:7899	Xanthomonas_oryzae	3.25E-61	51.83
GE003490	PHI:7052	Streptococcus_mutans	1.70E-131	51.75
GE002728	PHI:10199	Escherichia_coli	6.62E-174	51.75
GE001865	PHI:4614	Edwardsiella_tarda	1.34E-124	51.73
GE001436	PHI:9356	Magnaporthe_oryzae	0	51.73
GE000629	PHI:501	Saccharomyces_cerevisiae	2.95E-45	51.66

GE000726	PHI:6314	Pseudomonas_syringae	9.86E-80	51.57
GE003487	PHI:8558	Staphylococcus_aureus	3.41E-152	51.57
GE004306	PHI:3266	Escherichia_coli	9.75E-109	51.48
GE001423	PHI:5369	Salmonella_enterica	5.96E-36	51.46
GE000224	PHI:8623	Neisseria_meningitidis	2.89E-74	51.43
GE001231	PHI:9821	Listeria_monocytogenes	3.49E-130	51.41
GE001241	PHI:7399	Erwinia_amylovora	1.11E-97	51.33
GE002059	PHI:9835	Botrytis_cinerea	8.48E-160	51.33
GE003306	PHI:7947	Klebsiella_pneumoniae	5.67E-117	51.31
GE002929	PHI:3914	Parastagonospora_nodorum	4.64E-171	51.25
GE001313	PHI:10104	Salmonella_enterica	5.25E-113	51.19
GE000337	PHI:10612	Acinetobacter_baumannii	1.08E-117	51.18
GE003099	PHI:7689	Helicobacter_pylori	0	51.12
GE001261	PHI:6184	Escherichia_coli	1.21E-147	50.90
GE001608	PHI:9011	Xanthomonas_oryzae	1.84E-68	50.87
GE001243	PHI:7411	Erwinia_amylovora	7.26E-79	50.85
GE000356	PHI:12232	Pseudomonas_aeruginosa	4.09E-159	50.81
GE002301	PHI:6308	Staphylococcus_aureus	4.92E-37	50.79
GE000498	PHI:3951	Xanthomonas_campestris	1.05E-40	50.75
GE000872	PHI:9599	Pseudomonas_aeruginosa	2.31E-75	50.74
GE001013	PHI:123517	Ralstonia_solanacearum	3.70E-24	50.63
GE001525	PHI:7511	Klebsiella_pneumoniae	3.26E-80	50.63
GE002314	PHI:123791	Francisella_tularensis	1.54E-108	50.62
GE001097	PHI:124303	Staphylococcus_aureus	6.65E-146	50.62
GE001715	PHI:123441	Xanthomonas_oryzae	4.89E-20	50.60
GE000329	PHI:7631	Pseudomonas_aeruginosa	7.48E-124	50.57
GE000413	PHI:3784	Pseudomonas_aeruginosa	3.21E-116	50.45
GE003891	PHI:6980	Salmonella_enterica	8.24E-26	50.42
GE000775	PHI:5422	Haemophilus_influenzae	5.34E-75	50.41
GE002639	PHI:3118	Pseudomonas_syringae	1.12E-75	50.22
GE000563	PHI:4038	Staphylococcus_aureus	0	50.16
GE004309	PHI:11234	Streptococcus_suis	2.12E-134	50.12
GE001263	PHI:10162	Burkholderia_thailandensis	2.15E-36	50.00
GE002242	PHI:3966	Xanthomonas_campestris	8.70E-91	50.00
GE003204	PHI:6190	Staphylococcus_aureus	5.45E-179	50.00
GE003595	PHI:123702	Pseudomonas_aeruginosa	8.97E-30	50.00
GE003857	PHI:8623	Neisseria_meningitidis	5.36E-86	50.00
GE003966	PHI:11882	Salmonella_enterica	3.29E-21	50.00

2. identification of secretion systems

Gene ID	Secretion System	Hit_seq_len	Hit_i_eval	Hit_score	Hit_profile_cov	Hit_seq_cov
GE000024	T6SSi_tssB	167	1.30E-55	185.5	0.969	0.934
GE000026	T6SSi_tssK	447	1.00E-134	447.7	0.998	0.989
GE000027	T6SSi_tssL	230	1.10E-20	72.2	0.836	0.77
GE000029	T6SSi_tssD	164	1.80E-55	185.5	0.988	0.976
GE000030	T6SSi_tssH	885	0	1214.9	0.995	0.979
GE000031	T6SSi_tssI	793	6.10E-141	468.8	0.972	0.75
GE000046	T6SSi_evpJ	86	1.10E-10	39.6	0.853	0.837
GE000049	T6SSi_tssM	667	2.90E-134	447.2	0.551	0.934
GE000050	T6SSi_tssA	534	6.10E-79	264.2	0.995	0.856
GE000052	T6SSi_tssF	585	2.20E-118	394.3	0.987	0.979
GE000053	T6SSi_tssG	362	2.20E-49	166.2	0.934	0.856
GE000054	T6SSi_tssJ	182	1.30E-31	107.1	0.942	0.874
GE000055	T6SSi_tssE	152	2.70E-28	96.4	0.87	0.743
GE000057	T6SSi_tssA	464	5.40E-44	149	0.908	0.901
GE000345	T4aP_pilC	400	3.40E-93	310.2	0.991	0.833
GE000346	T4aP_pilB	462	1.00E-148	493.2	0.919	0.823
GE000347	T4aP_pilA	152	2.00E-18	64.5	0.744	0.382
GE002631	T4aP_pilW	178	1.70E-10	39.2	0.575	0.388
GE002632	T4aP_fimT	157	8.10E-13	46.6	0.894	0.376
GE003044	T4aP_pilQ	424	7.70E-57	189.5	0.924	0.399
GE003047	T4aP_pilN	181	1.30E-11	42.9	0.986	0.779
GE003048	T4aP_pilM	269	4.90E-15	53.9	1	0.584
GE002699	T4aP_pilT	336	1.30E-119	397.2	0.953	0.97
GE001053	Flg_flgB	138	7.30E-34	114.8	0.992	0.964
GE001054	Flg_flgC	135	1.80E-48	162.5	0.993	0.963
GE001821	Flg_sctV_FLG	698	4.40E-286	948.5	0.996	0.96

GE001822	Flg_sctU_FLG	383	4.60E-128	425.1	0.997	0.896
GE001885	Flg_fliE	104	1.30E-27	94.1	1	0.981
GE001886	Flg_sctJ_FLG	574	6.60E-164	544.6	0.996	0.96
GE001889	Flg_sctN_FLG	454	9.90E-195	645.1	0.995	0.967
GE001894	Flg_sctQ_FLG	136	2.20E-34	115.3	0.987	0.544
GE001896	Flg_sctR_FLG	245	1.80E-91	303.2	1	0.824
GE001897	Flg_sctS_FLG	90	2.10E-33	112.3	0.989	0.978
GE001898	Flg_sctT_FLG	262	2.10E-73	245.1	0.996	0.989
GE001145	T6SSi_tssJ	165	1.10E-19	68.5	0.831	0.709
GE001146	T6SSi_tssK	449	1.20E-129	431	0.993	0.973
GE001147	T6SSi_tssL	413	3.20E-55	185.1	0.991	0.516
GE001148	T6SSi_tssM	1210	4.30E-232	771.1	0.989	0.931
GE001151	T6SSi_tssB	178	9.00E-62	205.5	0.963	0.876
GE001154	T6SSi_tssD	161	3.30E-32	109.8	0.988	0.963
GE001164	T6SSi_tssE	192	7.00E-22	75.6	0.986	0.792
GE001165	T6SSi_tssF	624	2.50E-164	546	0.998	0.992
GE001166	T6SSi_tssG	349	3.90E-79	264	0.946	0.908
GE001167	T6SSi_tssH	870	0	1247.2	0.999	0.986
GE001169	T6SSi_tssI	644	7.50E-148	491.6	0.844	0.801
GE001171	T6SSi_evpJ	1508	1.10E-06	26.7	0.516	0.032
GE001174	T6SSi_tssI	848	8.10E-148	491.5	0.846	0.613
GE001180	T6SSi_evpJ	1484	1.20E-06	26.6	0.505	0.03
GE002169	T1SS_mfp	390	4.50E-41	139.1	0.505	0.497
GE002170	T1SS_abc	715	5.20E-108	359.9	0.998	0.706
GE002171	T1SS_omf	452	5.80E-41	138.4	0.975	0.832

3. Identification of genomic islands

Genomic_island	Scaffold_id	Start	End	Length
Genomic_island_1	Contig00001	201526	224482	22957
Genomic_island_2	Contig00001	707124	723145	16022
Genomic_island_3	Contig00001	817416	836245	18830
Genomic_island_4	Contig00001	2011235	2016667	5433
Genomic_island_5	Contig00001	2120981	2132023	11043
Genomic_island_6	Contig00001	2641556	2670549	28994
Genomic_island_7	Contig00001	2759225	2769854	10630
Genomic_island_8	Contig00001	3241324	3267423	26100
Genomic_island_9	Contig00001	3270798	3278028	7231
Genomic_island_10	Contig00002	447994	465284	17291
Genomic_island_11	Contig00004	28203	52490	24288

4. Identification of secondary metabolite gene clusters

Type	From	To	Most similar known cluster	Similarity	Gene number
hserlactone,arylpolyene	1080211	1138970	arylpolyenes：Other	88%	61
terpene	25153	48714	carotenoid：Terpene	100%	28
NI-siderophore	178500	208863	desferrioxamine E：Other	100%	26
