# Supplementary material for: An Emerging Bacterial Leaf Disease in Rice Caused by Pantoea ananatis and Pantoea eucalypti in Northeast China
Source: Microorganisms. 2025 Jun 13;13(6):1376. doi: 10.3390/microorganisms13061376 (PMC12195282; doi:10.3390/microorganisms13061376)
Supplement: Supplementary file 1 [file microorganisms-13-01376-s001.zip › Table S6.pdf]

Table S6 Comparative analysis of secretion systems among *Pantoea ananatis*, *Pantoea eucalypti*, and *Xanthomonas oryzae* pv. *oryzae*

	P. eucalypti (GY78-10)		*Xoo* (PXO99A)		*P. ananatis* (PA13)	
Secretion System	Locus No.	Component No.	Locus No.	Component No.	Locus No.	Component No.
T1S	1	3	1	3	1	3
T2S	0	0	1	10	0	0
T3S	1	11	1	11	0	0
T4S	1	9	2	9/10	2	6/5
T5S	1	1	1	5	1	1
T6S	2	14	1	19	1	15
