# Supplementary figures and images for: An Emerging Bacterial Leaf Disease in Rice Caused by Pantoea ananatis and Pantoea eucalypti in Northeast China
Source: Microorganisms. 2025 Jun 13;13(6):1376. doi: 10.3390/microorganisms13061376 (PMC12195282; doi:10.3390/microorganisms13061376)

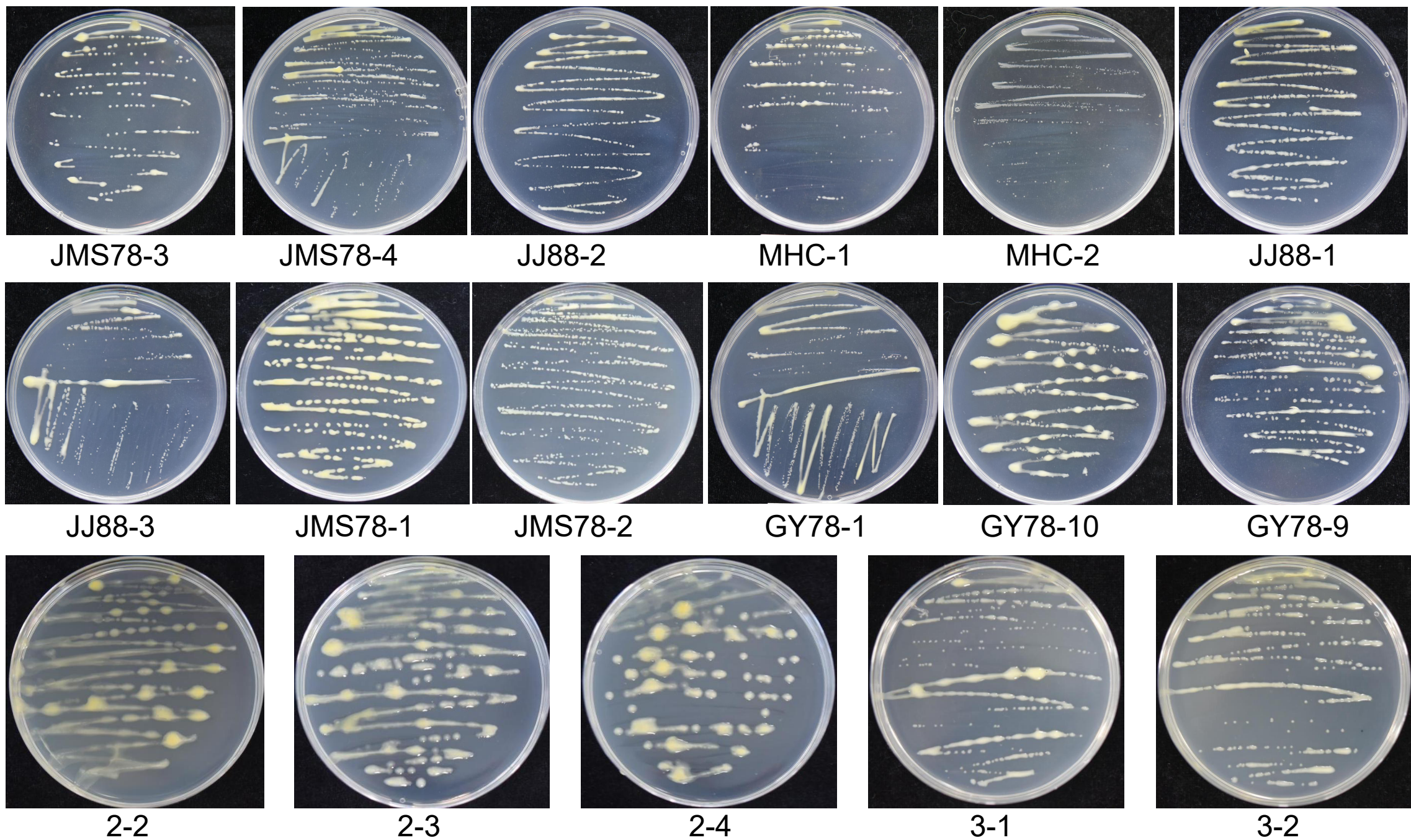

Figure S1. The colony morphological characteristics of 17 isolated strains.

Supplement: Supplementary file 1 [file microorganisms-13-01376-s001.zip › Figure S1.pdf]
